# A Fully Self‐Powered Digital Wearable System for the Auxiliary Treatment of Plantar Fasciitis

**DOI:** 10.1002/advs.202521682

**Published:** 2026-02-16

**Authors:** Jiacheng Hou, Ying Hong, Shiyuan Liu, Qiqi Pan, Jingyu Zhang, Qingyang Xu, Qiyi Nie, Zhonghe Wang, Liming Xin, Yilong Wang, Biao Wang

**Affiliations:** ^1^ Institute of Artificial Intelligence, School of Future Technology Shanghai University Shanghai China; ^2^ Department of Medical Ultrasound, National Clinical Research Center for Geriatrics West China Hospital, Sichuan University Chengdu China; ^3^ Thrust of Smart Manufacturing The Hong Kong University of Science and Technology (Guangzhou) Guangzhou China; ^4^ Department of Mechanical and Aerospace Engineering Hong Kong University of Science and Technology Hong Kong China; ^5^ School of Mechanical Engineering Hebei University of Technology Tianjin China; ^6^ School of Computer Engineering and Science Shanghai University Shanghai China; ^7^ School of Astronautics Harbin Institute of Technology Harbin China

**Keywords:** biomechanical, digital wearables, energy harvesting, flexible sensor, real‐time monitoring

## Abstract

Plantar fasciitis severely impairs daily life through persistent pain and limited mobility, whereas conventional treatments often lack real‐time monitoring and personalized feedback. This study introduces a *fully* self‐powered digital wearable system (FS‐DWS), integrating an arch support auxiliary (ASA) device, a wearable sensing system (WSS), and a machine learning‐driven closed‐loop visualized feedback system (VFS) to enable real‐time plantar pressure monitoring and abnormal gait recognition for the auxiliary treatment of plantar fasciitis. As a system‐level engineering achievement, the ASA module integrates elastic support with energy harvesting, alleviating plantar pressure and powering the wearable sensing system without any batteries, with a maximum power density of 41.6 mW/cm^3^, one order of magnitude higher than those of previously reported biomechanical energy harvesting devices. The VFS utilizes a flexible sensor array to collect dynamic pressure data, which is processed via a machine learning algorithm to achieve real‐time classification of 7 gait cycle phases with an accuracy of 99.3%, enabling identification of abnormal pressure distribution, causal tracing of gait deviations, and generation of personalized correction instructions. As a proof‐of‐concept study, the proposed dual‐function strategy of “physical support + intelligent regulation” provides an efficient and sustainable approach to the long‐term management of plantar fasciitis and supports a shift in therapeutic approach from passive relief to active correction.

## Graphical abstract

This study reports a system‐level fully self‐powered digital wearable system (FS‐DWS) for the auxiliary treatment of plantar fasciitis. By integrating arch support, energy harvesting, wearable sensing, and machine learning‐driven closed‐loop visualized feedback, the system enables effective plantar pressure reduction and self‐powered, real‐time plantar pressure monitoring and abnormal gait recognition, thereby transitioning therapy from passive relief to personalized rehabilitation.

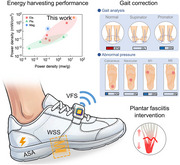

## 1 Introduction

Plantar fasciitis (also known as plantar aponeurosis) is the most common soft tissue foot disorder, primarily caused by arch collapse and excessive plantar pressure [1]. Studies show that plantar fasciitis affects 10% of U.S. adults and accounts for 25% of foot‐related conditions in athletes, with annual treatment costs exceeding $192 million [2, 3]. Owing to the persistent pain and restricted mobility it causes, plantar fasciitis not only disrupts daily life and work but can also cause joint abnormalities and structural damage to the foot [4]. Conventional treatments, including physical therapy, pharmacological interventions, orthotic support, and surgical procedures, [5] largely rely on static examinations by physicians and subjective feedback from patients [5, 6]. However, these methods lack the ability for real‐time dynamic monitoring and adjustment, leading to treatment outcomes that vary significantly across individuals and often delaying recovery [7]. Therefore, there is an urgent need for an innovative therapeutic solution capable of dynamically monitoring the recovery process, providing real‐time feedback, and adapting to individual patient needs in the management of plantar fasciitis.

With the rapid development of digital medical technology, wearable devices have demonstrated tremendous value in various disease management fields due to their capabilities of real‐time, continuous, and non‐invasive monitoring, [8, 9, 10, 11, 12, 13] such as the treatment and management of diabetes mellitus, [14, 15, 16, 17] the alleviation of sleep problems, [18, 19, 20] and the dynamic monitoring of physiological data using advanced self‐powered sensing architectures [21, 22, 23]. The pervasive application of wearable technology empowers individuals to better manage their own health, while providing medical professionals with comprehensive, real‐time data to facilitate more informed decision‐making. Regarding the field of foot‐related biomechanical monitoring and disease treatment, recent advancements include the exploration of wearable systems for foot movement monitoring and gait recognition, [24, 25, 26, 27, 28] foot nerve stimulation, [29, 30] foot pressure relief, [31, 32] and foot joint assistance [33, 34]. However, most current wearable devices for foot monitoring only focus on monitoring the movement status of the foot and do not provide specialized solutions for pathological foot problems such as plantar fasciitis. [35,36] In addition, a critical gap remains in the system‐level integration of energy harvesters with practical monitoring units. While energy harvesting is a known concept, most wearable energy recovery devices have insufficient output power under ultralow‐frequency motion [37, 38, 39] and cannot continuously supply power to the wearable sensing and monitoring system. Therefore, the innovation required extends beyond energy supply and necessitates a system‐level engineering breakthrough. Specifically, the simultaneous realization of high‐power energy harvesting and precise biomechanical monitoring—without relying on external power—remains a significant engineering bottleneck for constructing a closed‐loop auxiliary treatment system [40, 41, 42, 43].

In this study, we present a system‐level innovation through the development of a fully self‐powered digital wearable system (FS‐DWS) that is composed of an arch support auxiliary (ASA) device, a wearable sensing system (WSS), and a machine learning‐driven closed‐loop visualized feedback system (VFS), constructing a closed‐loop system including full energy self‐circulation, dynamic support, intelligent monitoring, and feedback intervention, for the auxiliary treatment of plantar fasciitis. The ASA module combines elastic support with energy harvesting, achieving the alleviation of plantar pressure and the conversion of mechanical energy from arch deformation during walking into electrical energy simultaneously; The WSS, composed of a flexible pressure sensor array and a signal processing system, and powered by the energy harvested from the ASA, collects the dynamic pressure data from human foot and performs data processing and wireless transmission; The VFS receives the dynamic pressure data through a signal receiving module, which is further processed via a machine learning algorithm to achieve real‐time classification of 7 gait cycle phases (99.3% accuracy), enabling identification of abnormal pressure distribution, causal tracing of gait deviations, and generation of personalized correction instructions. The ASA module features a highly optimized transmission mechanism that efficiently converts low‐frequency walking impact into high‐frequency rotation. Furthermore, by integrating sensors with a convolutional neural network (CNN) algorithm, the system achieves a high accuracy in gait phase classification. This enables the VFS to translate raw sensor data into interpretable feedback, upgrading the treatment logic from passive relief to active, personalized correction. Compared with existing devices, the developed FS‐DWS realizes the integration of fully self‐powered supply and dynamic support, the coupling of multi‐modal sensing and intelligent algorithms, and the upgrade of treatment logic from “passive relief” to “active correction”.

## 2 Results

### 2.1 Design and Working Mechanism of the FS‐DWS

**FIGURE 1:**
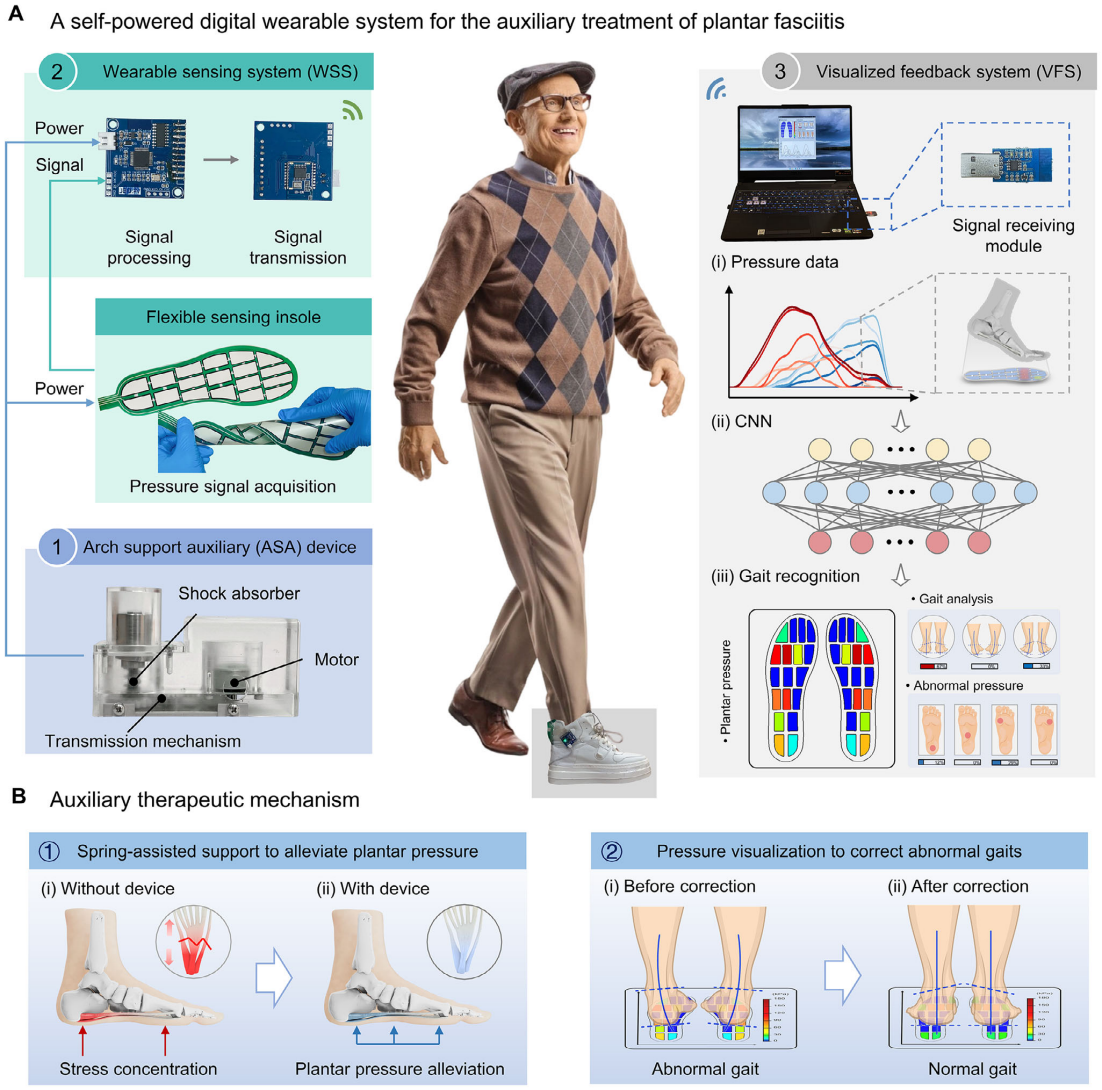
Design and working mechanism of the FS‐DWS. (A) FS‐DWS is a fully self‐powered wearable digital auxiliary treatment system for closed‐loop rehabilitation intervention of plantar fasciitis. (1) ASA device for elastic support and energy harvesting, achieving the alleviation of plantar pressure and the conversion of mechanical energy from arch deformation during walking into electrical energy simultaneously. (2) Flexible sensing insole, consisting of 18 flexible sensing units, collects plantar pressure signals in real time; Signal processing system transmits the collected signals to the signal processing module, and then realizes real‐time data transmission from the wearable device to the client through the signal sending module and the Bluetooth receiving module. (3) The gait recognition system integrated into the VFS constructs a database for training a CNN model using data acquired from wearable sensors. Once trained, the model is capable of classifying the input data and generating recognition results, realizing the real‐time visualization of plantar pressure distribution and abnormal gait identification. Note: Schematic illustration of an elderly gentleman walking. Reproduced with permission from LJSphotography/Alamy (Image ID:2GY8C0N). (B) Dual‐function strategy of “physical support + intelligent regulation” for the synergistic auxiliary treatment of plantar fasciitis. (1) The physical support from the ASA device redistributes peak pressures in the heel and metatarsal regions, thereby reducing stress on the plantar fascia and mitigating inflammatory stimuli. (2) The gait recognition system provides an intelligent regulation of the abnormal postures, achieving an active behavioral correction for precise intervention of foot pathology.

As shown in Figure 1A and Figure S1, the developed FS‐DWS consists of an ASA device for energy harvesting and pressure relief, a WSS for signal collection and processing, and a VFS for plantar pressure monitoring and gait recognition (Table S1). We design the ASA based on the biodynamics of human walking, which is composed of a limit frame, a transmission mechanism, a compression spring, and a brushless generator (Figure S2). Furthermore, the device is engineered with a compact profile to fit within the shoe heel, featuring an overall height of 48 mm and a width of 40 mm (detailed geometric parameters can be found in Figures S3 and S4). The compression spring with appropriate stiffness can support the navicular area of the sole to prevent arch collapse, relieve plantar pressure, and help alleviate plantar fascia pain and inflammation. Meanwhile, the transmission mechanism converts the low‐frequency linear motion from human walking into high‐frequency rotational motion, realizing the conversion of human kinetic energy into electrical energy through the integrated brushless generator. The generated electrical energy is then utilized to supply power to the WSS (i.e., the flexible sensing insole and the signal processing and transmission module) through the power management module (Figure S5), enabling the fully self‐powered function of the wearable device. As the sensing module of the WSS, the flexible sensing insole, consisting of 18 flexible sensing units, collects plantar pressure signals in real time, transmits them to the signal processing module, and then realizes real‐time data transmission from the wearable device to the client through the signal transmission module and the Bluetooth receiving module for data storage, analysis, and visualization. The fabricated prototype of the ASA device is presented in Figure S6. Through real‐time data monitoring, the wearable system composed of ASA and WSS significantly reduces patients’ reliance on conventional bulky rehabilitation equipment, thereby overcoming the limitations of traditional medical equipment in terms of time and space.

The gait recognition system integrated into the VFS constructs a database for convolutional neural network (CNN) model training using data acquired from the WSS. Once trained, the model is capable of classifying the input data and generating recognition results. In contrast to previous foot monitoring studies, [44] the developed gait recognition system enables the visualization of plantar pressure distribution, making users aware of specific foot conditions and thereby raising health self‐awareness and increasing engagement in rehabilitation exercises. Moreover, by leveraging the VFS and CNN‐based algorithms to analyze plantar pressure data, the system achieves abnormal gait identification and early detection of irregular pressure patterns. Ultimately, it generates personalized gait correction feedback reports for patients, which contributes to an accelerated rehabilitation process.

The energy harvesting and pressure relief capabilities of the ASA device, combined with the real‐time monitoring and feedback functions of the CNN‐based gait recognition system in VFS, exhibit synergistic complementarity in the auxiliary treatment of plantar fasciitis (Figure 1B). On the one hand, this biomechanically‐informed wearable device directly addresses the pathological mechanisms of plantar fasciitis (e.g., arch collapse and excessive pressure) through structured arch support. It physically redistributes peak pressures in the heel and metatarsal regions, thereby reducing strain on the plantar fascia and mitigating inflammatory stimuli, which establishes a foundation for pain alleviation and functional restoration. On the other hand, the gait recognition system translates imperceptible abnormal gait patterns into visually interpretable feedback via CNN algorithms, enabling dynamic tracking and precise intervention of foot pathology. This system not only provides timely alerts regarding abnormal pressure distribution—helping patients avoid gait patterns that could exacerbate injury—but also facilitates active behavioral correction through personalized reports. Thereby, the therapeutic approach is advanced from passive symptom management to active functional rehabilitation.

The proposed dual‐function strategy of “physical support + intelligent regulation” not only overcomes the limitations of conventional treatments—such as the absence of real‐time monitoring and insufficient personalization—but also accelerates the rehabilitation process through a closed‐loop intervention mechanism. It provides an efficient and sustainable approach to the long‐term management of plantar fasciitis, and demonstrates unique value in enhancing therapeutic outcomes and improving patients’ quality of life.

### 2.2 Biomechanical Analysis of Walking with the Use of the ASA

**FIGURE 2:**
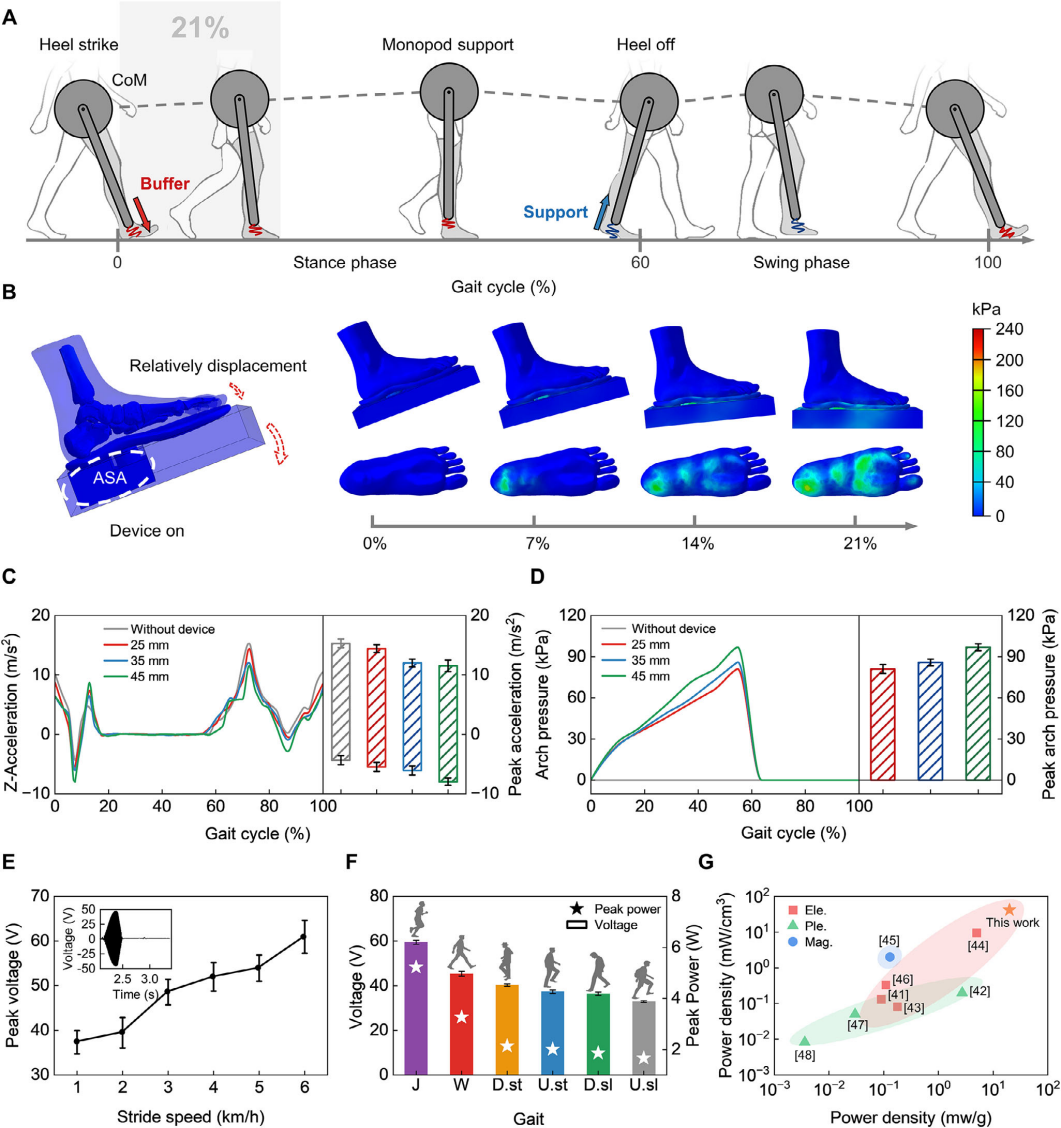
Biomechanical analysis and energy harvesting performance of the ASA device. (A) Dynamic changes of the ASA device during a gait cycle. The spring is connected to the transducer of the sole. When the foot touches the ground, the coil spring inside the sleeve is compressed, which plays a role in shock absorption and pressure sharing, preventing excessive peak plantar pressure. During the propulsion phase at the end of the stance phase, the recoil force of the spring assists the contraction of the calf muscles to achieve plantar flexion of the foot. (B) Biomechanical simulation shows the stress variations of plantar pressure during the touchdown process when wearing the ASA device, demonstrating that the physical support from the ASA provides adequate supportive force to the arch and achieves sufficient plantar cushioning effects. (C) Acceleration amplitude variations of the arch within a gait cycle when wearing the device with 3 different spring stiffnesses and when not wearing the device. (D) Arch pressure variations within a gait cycle. The average step cycle is 1.24 ± 0.02 s. Each participant (N = 7) conducts a 5 min treadmill walking test under each condition. (E) Peak voltage of the ASA device at different stride speeds. Insets show the voltage waveform within a gait cycle. (F) Peak voltage and peak power of the ASA device under different gait conditions, including jogging (J), walking (W), downstairs (D.st), upstairs (U.st), downslope (D.sl), and upslope (U.sl). (G) Comparison of the power density between the proposed ASA device and previously reported biomechanical energy harvesting devices.

Gait, as a manifestation of the movement pattern of the lower limbs during human walking, is closely related to the biomechanical characteristics of the foot (e.g., plantar pressure distribution and joint force). In this study, we employ a simplified model of human walking—the inverted pendulum model—to elaborate on the impact of the ASA on human walking biomechanics and investigate how spring stiffness affects walking motion (Figure 2A; Figure S7). In this model, the foot moves the center of mass (CoM) as an inverted pendulum. The collision between the sole and the ground is considered in the model at the moment of contact, and the dynamics at the instant of contact can be described by the impulse theorem: the time integral of the collision force equals the change in body momentum during the early stance phase. During this phase, the lower limbs stop abruptly, while other parts of the body decelerate gradually (detailed description can be found in Text S1). Throughout this movement process, the model demonstrates the biodynamics of vibration damping and gait assistance during the initial and early stance phases, respectively (Figure 2A; Figure S8). The observed spring‐like response of the plantar tissue under loading during gait indicates that supplemental support, provided through artificial springs, may be effective in mitigating elevated plantar pressure. Biomechanical simulation analysis demonstrates that the physical support from the ASA can provide adequate supportive force to the arch (detailed parameters can be found in the Tables S2–S5), achieving sufficient plantar cushioning effects (Figure 2B; Figures S9 and S10). Based on model predictions and the mechanical properties of plantar tissues, we estimate that the suitable range of supportive pressure required for the arch is 80–100 kPa.

For verification, we formulate an experimental gradient of initial supportive forces by testing springs with different free lengths (25, 35, and 45 mm), with corresponding stiffnesses of 10.00, 7.14, and 5.56 N/mm, respectively. Given the fixed installation space within the ASA device (approximately 10 mm height), and considering the minimum thrust required for device reset, these varying lengths induce different levels of pre‐compression (preload), thereby modulating the initial arch support intensity. We investigate the supportive force in the navicular bone region and the acceleration amplitude during landing when human subjects wear the ASA device equipped with these springs of varying lengths (Figure S11). This is aimed at identifying the optimal preload configuration length to provide appropriate supportive force (consistent with the simulation target of 80–100 kPa) to the navicular bone area. As shown in Figure 2C, the ASA increases the acceleration amplitude of the arch during landing and reduces the acceleration amplitude of the arch during the take‐off phase, indicating that wearing the ASA device plays a cushioning role during the landing phase and a propelling role during the take‐off phase. Figure 2D shows that the ASA device can provide supportive force to the arch when walking, thereby dispersing the peak pressure on the plantar region. Moreover, no significant differences are observed in hip, knee, or ankle joint angles, nor in step length when walking with and without the ASA device, confirming that the proposed ASA device does not interfere with normal joint biomechanics or impede natural walking patterns (Figures S12 and S13). Considering the driving force of the energy harvesting device and wearing comfort, we select a 25 mm spring to provide supporting force.

### 2.3 Energy Harvesting Performance of ASA

Besides plantar pressure relief, we further investigate the energy harvesting performance of the ASA device under six common gaits (i.e., jogging, walking, downstairs, upstairs, upslope, downslope). As shown in Figure 2E, the open‐circuit voltage of the ASA device increases accordingly with the increase in stride speeds. In addition, the output voltage varies for different gait patterns, due to the differences in dynamic features between gait patterns (Figure 2F; Figure S14, and Text S2). Among the six gaits, the ASA device produces a maximum power output during jogging, with a peak open‐circuit voltage of 59.3 V and a peak power of 5.22 W (Figure S15). Compared to existing electromagnetic, piezoelectric, and magnetofluid foot energy harvesters (Table S6), our ASA device achieves a superior peak power density of 20 mW/g and 41.6 mW/cm^3^ (Figure 2G), representing an order‐of‐magnitude improvement in volumetric energy density over previously reported biomechanical energy harvesting devices [45, 46]. This high performance is attributed to the optimized transmission mechanism that efficiently converts low‐frequency walking impact into high‐frequency rotation, addressing the power sufficiency issue commonly encountered in wearable electronics. Owing to the high‐performance energy harvesting properties, the ASA device can charge capacitors of 2.2, 4.7, and 6.8 mF to 28.6, 25.0, and 24.4 V within 60s, respectively (Figure S16), and achieve fully self‐powered digital medical applications for wearable devices. When walking at a normal speed, the power output generated by ASA can power two 3 W light‐emitting diode bulbs (Movie S1 and Figure S17), a wearable watch (Movie S2). Besides, the system's robustness is validated across participants with varying body mass, as detailed in Figure S18. Even for the user weighing 54 kg, the output remains sufficient for the WSS operation (Text S3 and Movie S3). Moreover, the ASA device can still provide a stable power supply without obvious attenuation after nine months (Figure S19). These demonstrations exhibit that the ASA, as a mobile self‐generating device, is feasible for powering outdoor wearable smart devices, offering a new solution for achieving full self‐powering of wearable devices.

### 2.4 Visualization of Plantar Pressure and Pressure Alleviation Mechanism

**FIGURE 3:**
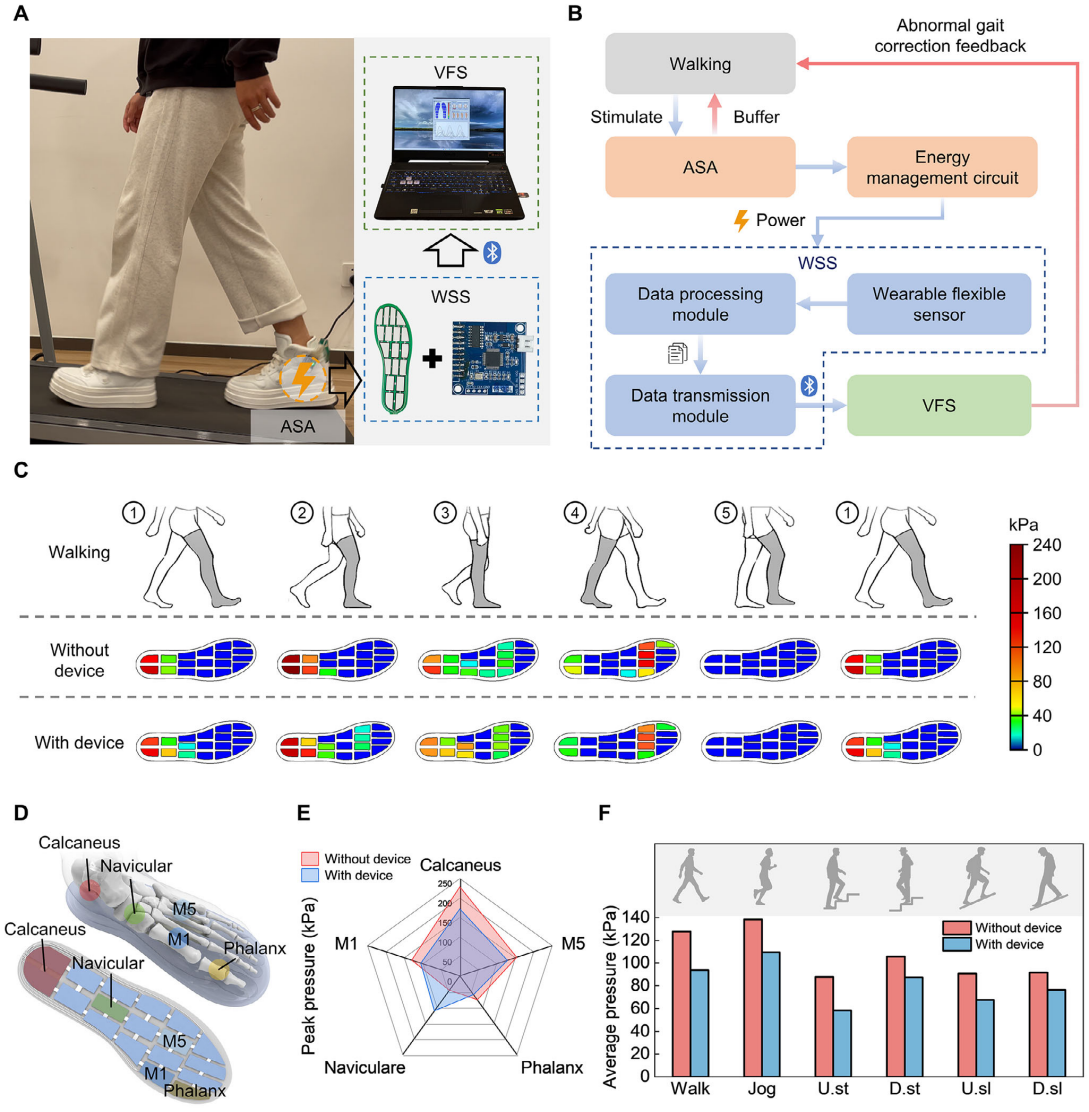
Visualization of plantar pressure and pressure alleviation mechanism. (A) The ASA device supplies power for the wearable sensor array and the wireless data transmission module through an energy management circuit. (B) Working mechanism of the developed FS‐DWS. During user walking, the ASA device alleviates plantar pressure and converts kinetic energy into electrical energy to power the wearable sensing system. Within this system, flexible sensors acquire plantar pressure signals, which are processed by a data acquisition module and wirelessly transmitted to a terminal of a gait recognition system via Bluetooth. The gait recognition system utilizes a CNN model to perform gait analysis and provides real‐time feedback on detected abnormalities. (C) Dynamic plantar pressure distributions within a gait cycle when wearing and not wearing the device, respectively. (D) Selected sensor units of the insole for pressure distribution analysis corresponding to the five key regions of the foot. (E) Pressure variations in the five key regions when wearing and not wearing the device. (F) Variations of the average peak pressure in the key regions except the navicular region under six representative gaits when wearing and not wearing the device. The pressure decreases in these regions indicate that an appropriate arch support force from the ASA device can effectively relieve plantar pressure under multiple gaits, which is highly beneficial for alleviating plantar fasciitis.

The kinetic energy generated by the ASA device during human walking is utilized to supply power for the wearable sensor array and the wireless data transmission module in the WSS through an energy management circuit, enabling fully self‐powered wireless transmission of sensing data (Figure 3A; Figure S20). Figure 3A illustrates the working mechanism of the developed FS‐DWS. During user walking, the ASA device simultaneously converts kinetic energy into electrical energy and alleviates plantar pressure. Concurrently, the collected electrical energy is used to power the WSS via an integrated energy management circuit. Within this system, flexible sensors acquire plantar pressure signals, which are processed by a data acquisition module and wirelessly transmitted to a terminal with a gait recognition system via Bluetooth. The gait recognition system in VFS utilizes a CNN model to perform gait analysis and provides real‐time feedback on detected abnormalities. This integrated process helps prevent incorrect gait patterns and excessive loading during rehabilitation, thereby facilitating the recovery of plantar fasciitis. Furthermore, by offering visual real‐time feedback on plantar pressure distribution, the system enhances users’ awareness of their musculoskeletal health and promotes active engagement in rehabilitation training.

As shown in Figure 3C, the plantar pressure data of a walking gait cycle with and without the ASA device are visualized in the VFS. Five key regions, including calcaneus (Cal.), navicular (Nav.), first metatarsal (M1), fifth metatarsal (M5), and phalanx (Pha.) regions, are selected as representative areas for dynamic pressure, which can centrally reflect the plantar weight‐bearing patterns (Figure 3D; Figure S21). Thermal and latency evaluations confirm system reliability. The WSS maintains stable temperatures during 10 min walking tests (Figure S22 and Movie S4). The excellent thermal stability ensures user comfort and prevents thermally induced degradation of energy harvesting efficiency during long‐term operation. The system's robustness is validated across participants with varying body mass and height (Figure S23). Previous studies [47] have shown that during human movement, the calcaneus region often bears the greatest pressure and is most prone to abnormal pressure. Figure 3E shows the peak pressures in the five key regions when walking with and without the ASA device. The experimental results indicate that when users walk with the ASA, the ASA supports the navicular region, disperses plantar pressure, and reduces the pressure in other regions, thereby relieving the tension on the plantar fascia. A decrease from 16.71% to 33.64% of the peak average plantar pressure is achieved in six representative gaits (Figure 3F; Figures S24–S30, and Table S7). The total pressure in the calcaneus and metatarsal regions decreases under various gaits, with a significant reduction in calcaneus pressure, indicating that an appropriate arch support force from the ASA device can effectively relieve plantar pressure under multiple gaits, which is highly beneficial for alleviating plantar fasciitis.

### 2.5 Machine Learning‐driven Gait Recognition

**FIGURE 4:**
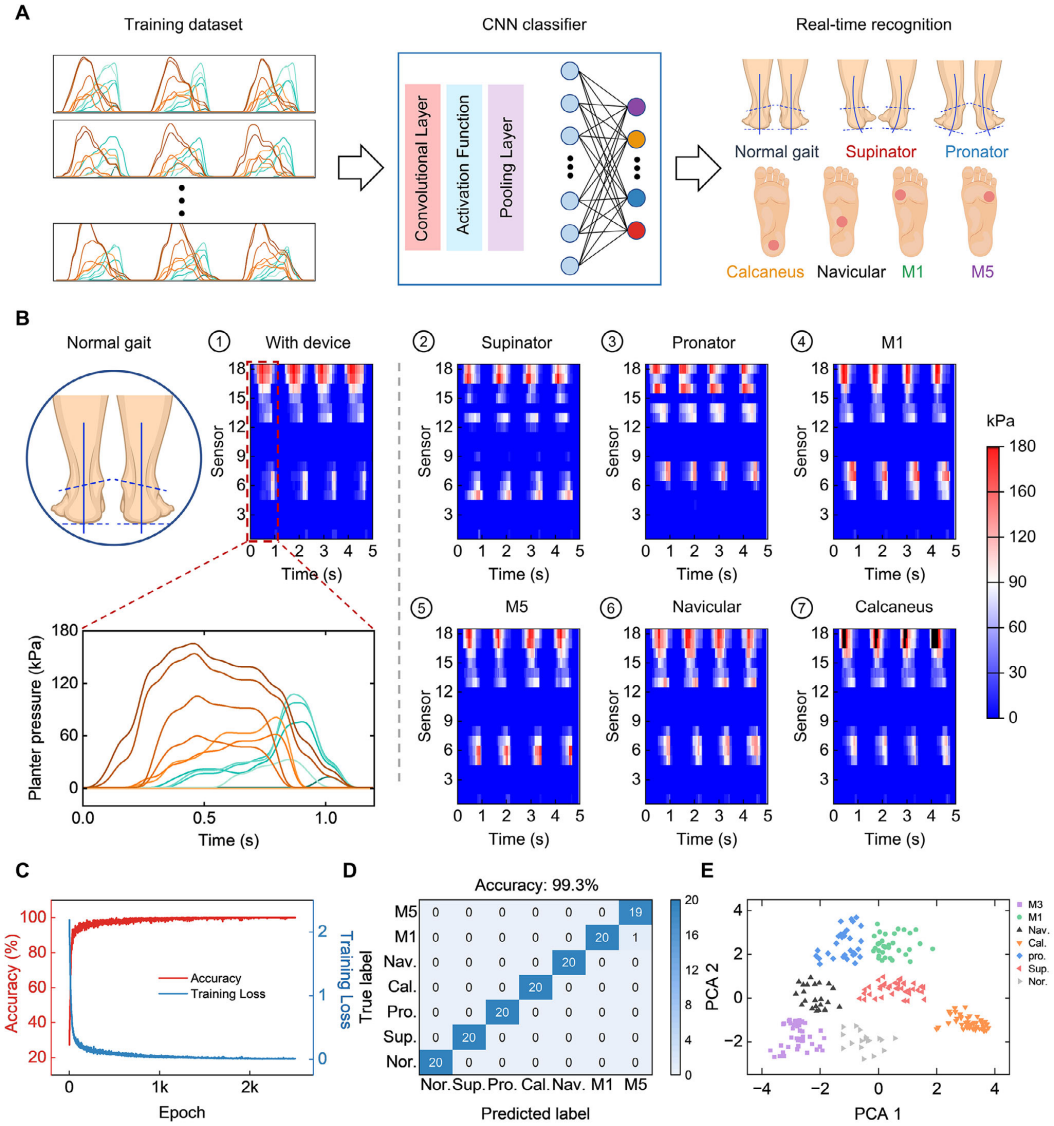
Machine learning‐driven gait recognition system. (A) Workflow diagram of the gait recognition learning model, including data collection, CNN model training, and gait classification output. (B) Representative data of seven gait patterns for CNN model training, including normal gait, supinator, pronator, and abnormal pressures in heel, arch, M1, and M5. Data are collected at a frequency of 20 Hz. Inset shows the plantar pressure signals from the 18 flexible sensors in the insole. (C) Training accuracy of the CNN algorithm‐based model and loss of gait recognition. (D) Prediction confusion matrix of the CNN algorithm, showing an accuracy of 99.3%. (E) PCA visualization results of data corresponding to the seven gait patterns, where data points of motion states cluster in distinct regions, significantly reducing the difficulty of classification.

When a user wears the fully self‐powered wearable system, data is transmitted to the VFS in real time, and the VFS uses a CNN model to classify and identify different human gaits. Figure 4A outlines the main workflow. Plantar pressure data with spatiotemporal characteristics are first collected under seven representative gait patterns, including normal gait, supinator (Sup.), pronator (Pro.), and abnormal pressures in heel, arch, M1, and M5 (Figure S31). Specifically, for each gait pattern, we collect 40 data samples (each containing time signal and pressure signals from effective channels), resulting in a total dataset of 280 samples. These samples are then used to train the CNN model. After training, the model can classify input data and generate recognition results. The CNN classification model is specifically designed to process the time‐series pressure signals. It consists of two sequential convolutional layers with kernel sizes of 2 × 1 and channel depths increasing from 16 to 32 for feature extraction, each followed by a batch normalization layer and a Rectified Linear Unit (ReLU) activation function. A max‐pooling layer is inserted to reduce dimensionality, while a fully connected layer maps the extracted features to the output classes. Finally, a softmax layer outputs the probability distribution for the 7 gait categories (detailed information can be found in Figure S32 and Table S8).

Figure 4B displays representative data collected under the seven gaits, presented as heatmaps with a duration of 5 s. During normal human walking, plantar pressure is reasonably and evenly distributed; in cases of the supinator and pronator, plantar pressure tends to shift to one side, resulting in pressure imbalance; when there are pressure abnormalities in heel, arch, M1, or M5, local pressure increases abnormally (Figures S33–S35). With the increase in the number of iterations, the training loss of the CNN model decreases steadily, and the training accuracy gradually improves until final convergence is achieved, with the model attaining an excellent recognition accuracy of 100% (Figure 4C). The prediction confusion matrix of the CNN algorithm in Figure 4D shows an accuracy of 99.3%. This is further confirmed by Principal Component Analysis (PCA) in Figure 4E, where data points of motion states cluster in distinct regions, significantly reducing the difficulty of classification. These results clearly validate the reliability and accuracy of the developed VFS.

### 2.6 Closed‐Loop Real‐Time Monitoring and Feedback for Active Gait Correction

**FIGURE 5:**
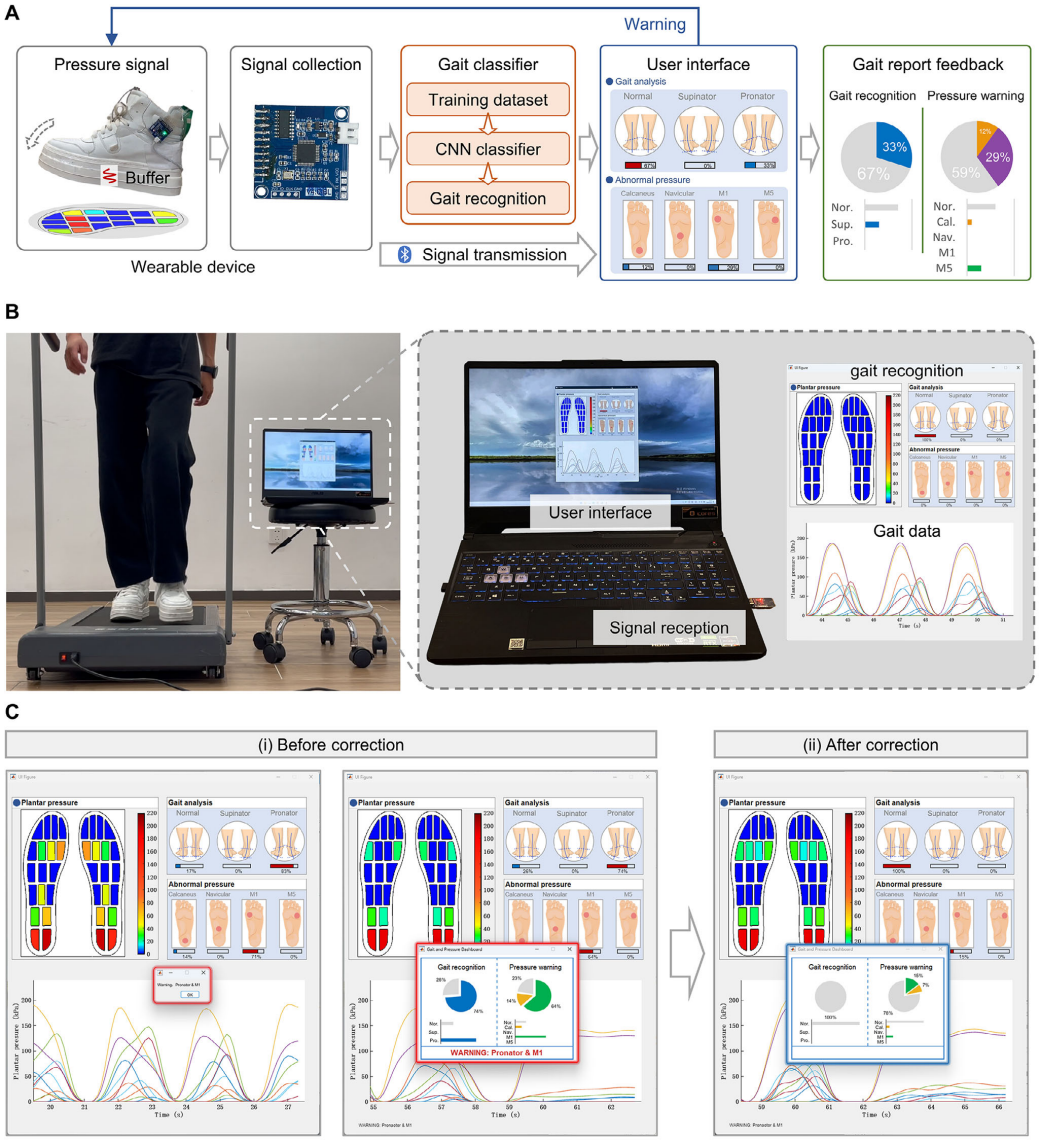
Closed‐loop design of the VFS with real‐time monitoring and feedback for active gait correction. (A) Working flow of the closed‐loop system. The wearable flexible sensors record plantar data in real time and transmit it to the user terminal. The VFS performs data analysis based on CNN to distinguish between normal gaits and abnormal pathological gaits, and conducts real‐time identification, early warning, and intervention on abnormal gaits and pressure points during human walking. Finally, it generates a comprehensive gait feedback report to track rehabilitation progress and facilitate gait correction for users. (B) Optical image of the visualized terminal user interface of the gait recognition system. (C) Real‐time display of the VFS user terminal interface when abnormal gaits occur during human walking. (i) When the system detects an abnormal gait, a pop‐up window appears for real‐time warning, and a gait recognition feedback report is provided for the entire walking cycle after walking ends. (ii) Once the user successfully corrects the abnormal gaits, the user receives a feedback report reflecting the corrected gaits without further warning on abnormal gaits.

Conventional treatment methods lack real‐time monitoring of patients’ rehabilitation progress and dynamic feedback, leading to significant fluctuations in treatment outcomes due to individual differences. Moreover, users may still exhibit incorrect gaits and postures during foot correction, which slows down the recovery process and may even exacerbate the condition. Therefore, we construct a VFS with real‐time correction and feedback, as shown in Figure 5A. In this system, wearable flexible sensors record plantar data in real time and transmit it to the user terminal. The VFS performs data analysis based on CNN to distinguish between normal gaits and abnormal pathological gaits (i.e., supinator and pronator), as well as to identify abnormal plantar pressures (i.e., abnormal pressure in heel, arch, M1, and M5). It provides real‐time early warning and reminders for gait abnormalities and aberrant pressure points during walking. Subsequently, it generates a comprehensive gait feedback report to track rehabilitation progress and facilitate gait correction for users.

As shown in Figure 5B, when the user wears the VFS during walking, the system displays a real‐time and visualized user interface on the computer screen, which is used for intuitive gait recognition and early warning. Figure 5C, Figure S36 and Movie S5 demonstrate the real‐time display of the VFS user terminal interface when abnormal gaits occur during human walking. When an abnormal gait (e.g., pronator) or an abnormal pressure point (e.g., M1) appears during walking, a pop‐up window appears on the user terminal interface for real‐time warning, reminding the user to correct the corresponding abnormal gait; after the user finishes walking, a gait recognition feedback report for the entire walking cycle appears on the user terminal, which is used for the user's self‐diagnosis and as data support for subsequent treatment, accelerating the recovery process of plantar fasciitis. Once the user successfully corrects the abnormal gaits based on the previous gait analysis report, no more pop‐up windows will appear on the user terminal during walking, and the user will receive a feedback report reflecting the corrected gaits without further warning on abnormal gaits and pressure points.

## 3 Discussion

Plantar fasciitis, a chronic foot disorder characterized by plantar pain and limited mobility, often features a recurrent and protracted course. This makes daily refined monitoring and continuous care crucial for influencing rehabilitation outcomes [48]. However, current treatment methods have significant shortcomings in meeting such dynamic rehabilitation needs. In conventional approaches, like physical therapy, pharmacological interventions, and custom orthotics, the user's rehabilitation process lacks dynamic feedback, and the treatment plan cannot be adjusted in a timely manner, resulting in poor treatment effects. Additionally, most existing wearable devices for foot rehabilitation rely on batteries for powering components such as signal processing units, failing to meet energy demand for full system operation and remaining at the monitoring level and lacking appropriate follow‐up feedback and intervention measures. Therefore, developing an innovative auxiliary treatment system capable of real‐time dynamic monitoring, providing personalized feedback and intervention, and operating without any external energy supply is of great practical significance and urgency for breaking through the existing treatment bottlenecks of plantar fasciitis and meeting patients’ needs for daily refined monitoring and continuous care.

In this study, we demonstrate a system‐level fully self‐powered FS‐DWS, providing an innovative solution for the auxiliary treatment of plantar fasciitis. Through the synergistic effect of the ASA, the WSS, and the machine learning‐driven VFS for plantar pressure recognition and warning, the system constructs a closed‐loop treatment including complete energy self‐circulation, dynamic support, intelligent monitoring, and feedback intervention. The ASA converts the mechanical energy generated by arch deformation during walking into electrical energy to realize the fully self‐powered supply of the wearable sensors and the signal transmission module, with a maximum power density of 41.6 mW/cm^3^, far exceeding the results previously reported. In the meantime, it also provides cushioning during the landing phase and propulsive force during the take‐off phase through elastic support, effectively dispersing plantar pressure. The ASA device reduces the peak plantar pressure by 16.71%–33.64% in six common gaits without affecting normal joint functions and daily activities. The VFS utilizes a distributed sensor array in the WSS for dynamic plantar pressure data acquisition, which is subsequently processed via a CNN model, achieving a gait recognition accuracy of 99.3%. The system enables real‐time monitoring of plantar pressure distribution, gait analysis, early detection of abnormalities, and personalized corrective feedback reports, supporting a shift in therapeutic approach from passive symptom relief to active gait correction.

It should be noted that the current evaluation is conducted on healthy participants who simulate abnormal gaits, rather than patients with active plantar fasciitis. While this study successfully validates the engineering feasibility (full energy autonomy) and biomechanical mechanism (pressure redistribution) of the FS‐DWS, the complex kinematic compensations caused by chronic pain (antalgic gait) in actual patients may introduce variables not fully captured by healthy subjects. Therefore, this work represents the preliminary validation phase, focusing on establishing the system's “physical support + intelligent regulation” capabilities.

In conclusion, the FS‐DWS distinguishes itself through a system‐level engineering achievement that bridges the gap between energy harvesting and practical application. The distinct advantages of the FS‐DWS over existing solutions can be summarized as follows: (1) 100% energy autonomy: Unlike devices that rely on batteries or low‐power harvesters, the ASA delivers a record‐high power output. This exceptional output enables the integration of reliable sensing components into the WSS, demonstrating a practical engineering pathway to overcome the energy bottleneck in wearable systems. (2) Synergistic therapy: It uniquely integrates passive physical support (reducing peak pressure by up to 33.64%) with active intelligent regulation, addressing both the symptoms (pain) and the root cause (abnormal gait). (3) Closed‐loop intervention: By establishing a real‐time feedback loop from sensing to visualization, it empowers patients to actively correct their gait, a feature often absent in conventional orthotics. Future work will focus on bridging the gap between this engineering proof‐of‐concept and clinical application through longitudinal trials involving diagnosed populations, aiming to quantify long‐term therapeutic outcomes such as pain reduction scores and tissue healing rates.

## 4 Methods

### 4.1 Participants

Experiments are conducted with seven healthy male participants to evaluate the power generation performance of the FS‐DWS (Table S8). None of the participants reports any known or apparent injuries that could affect their gait. After explaining the nature of the study and its potential consequences, all participants provide written informed consent prior to their involvement.

### 4.2 Hardware Implementation

Shoes equipped with the ASA device are designed for the participants, and the effects of three compression springs with original lengths of 25, 35, and 45 mm are tested. The stiffness of the springs is measured based on the relationship between displacement and force using a load sensor. The wearable flexible sensing system is powered by the ASA, and data is transmitted to the client via Bluetooth. The client then processes and analyzes the data using serial communication with MATLAB. The ASA weighs approximately 300 g, with the ASA embedded in the shoe heel, the flexible sensors attached to the insole surface, and the circuit module mounted on the outer right side of the shoe. Three six‐axis attitude sensors are connected to the participant's thigh, calf, and the front right side of the shoe, with one additional six‐axis attitude sensor attached to the outer right side of the shoe to measure real‐time physiological data. To reduce the weight of the shoes, all components are made from lightweight materials. After adjusting for the mass of the heel removed to accommodate the ASA, the net weight increase of the shoes with the embedded device is negligible.

### 4.3 Experimental Procedure

The detailed experimental procedure is as follows: With the device powered on, participants wear the equipment and perform standing, walking, jogging, uphill walking, and downhill walking on a treadmill, as well as stair climbing and descending in a staircase. Each gait pattern lasts for 5 min, with biomechanical data recorded during the final 2 min. A 5 min rest is provided between sessions. The same conditions are used for data collection without wearing the device and under scenarios of abnormal gait and abnormal plantar pressure. These experimental conditions are selected to minimize the effects of fatigue caused by prolonged walking. The following data is recorded: arch pressure, acceleration amplitude, stride length, and changes in the angles of the hip, knee, and ankle joints while participants walk with and without the device. The energy harvesting performance of the ASA is measured across six gait patterns. Plantar pressure data is recorded during the participants’ movement across these six gait patterns, both with and without the device. The operational process of the WSS during walking is observed to validate its fully self‐powered functionality. Additionally, plantar pressure data is collected when participants wear the device under abnormal gait and abnormal pressure conditions. In the experiment, unless otherwise specified, the subject weighs 54 kg.

### 4.4 Testing Devices

Some gait experiments are conducted on a treadmill (HSM‐W03D, Healthmate Co., Ltd, China). A six‐axis attitude sensor (IMU948, Chenyi Electronic Technology Factory, China) is used to record acceleration amplitude during landing, joint angle changes, and stride length, with a sampling rate of 250 Hz. A plantar pressure sensor (RX‐ES42‐18, Changzhou Roxi Electronic Technology Co., Ltd, China) recorded arch pressure and plantar pressure, with a baud rate of 11200. A sensor data acquisition module (Guantuo Electronic Technology Co., Ltd, China) is used to read the data from the plantar pressure sensors. The collected data is transmitted to the client via a Bluetooth transmission module (CH340, Guantuo Electronic Technology Co., Ltd, China). The ASA outputs three‐phase electricity, which is converted into direct current through a rectifier bridge. The direct current is then supplied to capacitors (Hunan Aihua Group Co., Ltd, China) via a high‐power voltage regulation module (ZG1602, Chengdu Zhigong Electronic Technology Co., Ltd, China). The voltage generated by the ASA is recorded using an oscilloscope (Rohde & Schwarz, RTM3000, USA). A high‐resolution infrared thermal imager (HIKVISION H10, Hikvision Co., Ltd, China) is used to monitor the surface temperature of the device while a subject walks on a treadmill at a constant speed (1.5 m/s) for 10 min.

### 4.5 Data Processing

The inverse dynamics analysis is conducted to determine arch pressure, hip, ankle, and knee joint dynamics, arch acceleration amplitude, and plantar pressure across different gait patterns. The support provided by the ASA to the arch is evaluated by measuring arch pressure and acceleration amplitude during walking. The peak arch pressure is identified as the maximum value recorded during the stance phase (0–60% of the gait cycle). The electrical power output of the ASA is calculated as the ratio of the square of the external peak voltage to the load resistance over 20 consecutive gait cycles. To assess the feasibility of the FS‐DWS for individuals with varying body weights, the body weight of each participant is normalized for the analysis. Each gait in the database is a data sample consisting of a time signal and thirteen sets of channel pressure signals (five channels with very small pressure changes are excluded from the eighteen sets of channels). A total of 280 samples are collected by repeating each gait 40 times, and these samples are used to construct the corresponding dataset. To train the CNN model, 50% of the samples are randomly selected from each gait, while the remaining samples are used for testing purposes.

### 4.6 Statistics

For each condition, the mean and standard error of arch pressure, arch acceleration amplitude, joint kinematics of the hip, knee, and ankle, electrical power output, and plantar pressure are calculated across all participants. A paired *t*‐test is performed to identify conditions that caused significant changes in the biomechanical data. Statistical significance is defined as *p* < 0.05.

## Conflicts of Interest

The authors declare no conflict of interest.

## Supporting information




**Supporting File**: advs74472‐sup‐0001‐MovieS1.mp4

**Supporting File**: advs74472‐sup‐0002‐MovieS2.mp4

**Supporting File**: advs74472‐sup‐0003‐MovieS3.mp4

**Supporting File**: advs74472‐sup‐0004‐MovieS4.mp4

**Supporting File**: advs74472‐sup‐0005‐MovieS5.mp4

**Supporting File**: advs74472‐sup‐0006‐SuppMat.pdf

## Data Availability

The data that support the findings of this study are available in the supplementary material of this article.
