# Supplementary material for: A Fully Self‐Powered Digital Wearable System for the Auxiliary Treatment of Plantar Fasciitis
Source: Adv Sci (Weinh). 2026 Feb 16;13(24):e21682. doi: 10.1002/advs.202521682 (PMC13116193; doi:10.1002/advs.202521682)
Supplement: Supplementary file 6 — Supporting File: advs74472‐sup‐0006‐SuppMat.pdf [file ADVS-13-e21682-s001.docx]

Supplementary Materials for

**A Fully Self-Powered Digital Wearable System for the Auxiliary Treatment of Plantar Fasciitis**

Jiacheng Hou^#^, Ying Hong^#^, Shiyuan Liu^#^, Qiqi Pan^#^, Jingyu Zhang^*^, Qingyang Xu, Qiyi Nie, Zhonghe Wang, Liming Xin^*^, Yilong Wang^*^, Biao Wang^*^

# These authors contribute equally to this work.

* Corresponding authors. B. W. (biaowang6@shu.edu.cn); J. Z. (zhangjingyu_bf@hebut.edu.cn); L. X. (xinliming@shu.edu.cn); Y. W. (yl.wang@hit.edu.cn).

**This file includes:**

Figures S1 to S36

Tables S1 to S9

Texts S1 to S3

Captions for Movies S1 to S5

**Other Supplementary Materials for this manuscript include the following:**

Movies S1 to S5

**Supplementary Figures**


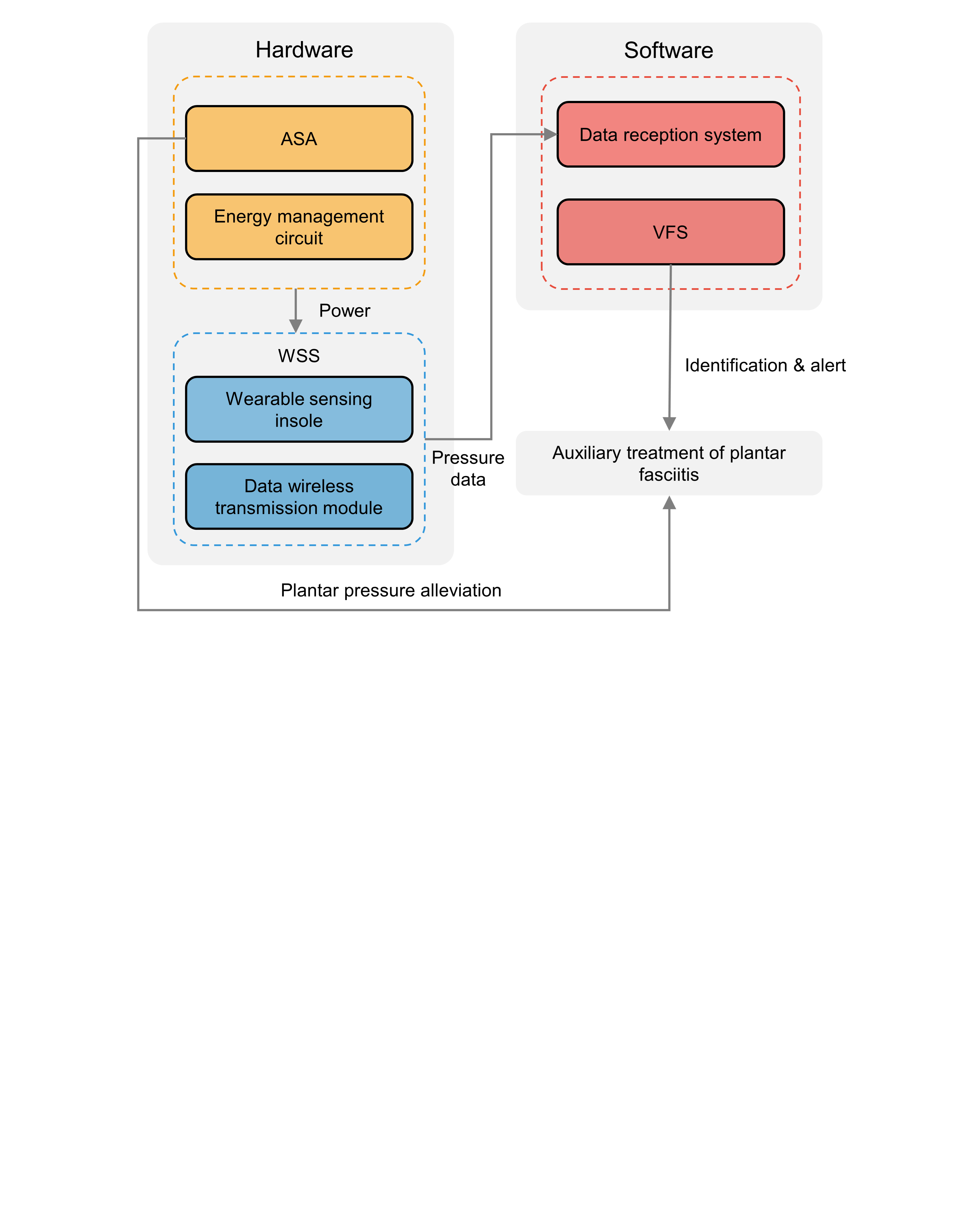


**Fig. S1.** **Composition of the FS-DWS system.** The hardware of FS-DWS consists of an ASA device, an energy management circuit, a wearable flexible sensor array, and a data wireless transmission module. The software is composed of a data receiving system and a VFS system.


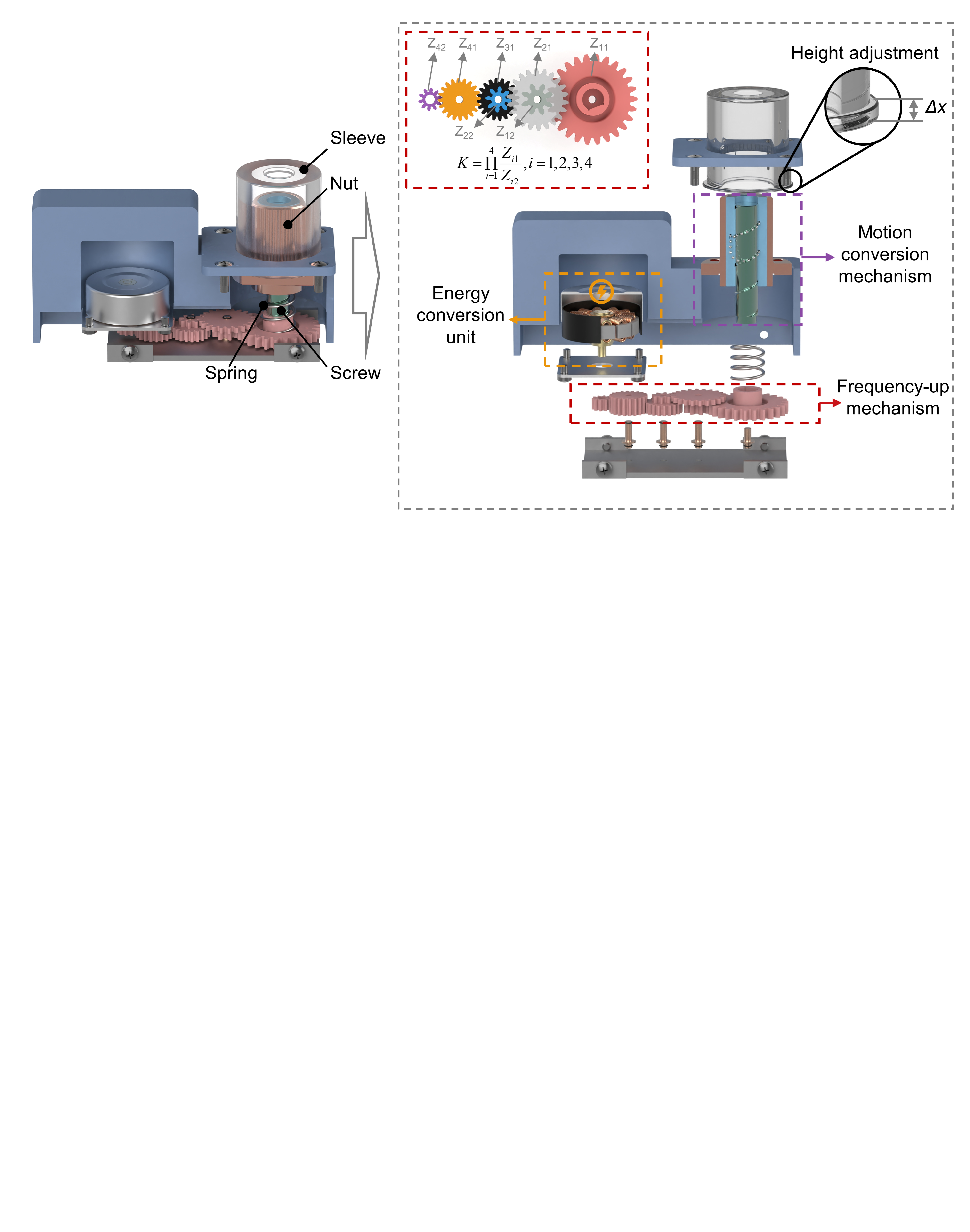


**Fig. S2. Composition of** **the ASA device.** The ASA is mainly composed of a sleeve, a ball screw transmission mechanism, a gear transmission mechanism, a compression spring, and a brushless motor. The edge thickness Δ*x* is used to adjust the support height of the device; the ball screw transmission mechanism is used to convert linear motion into rotational motion; the gear transmission mechanism is used to convert the low rotational speed of the input gear into the high rotational speed of the output gear; the compression spring is used to absorb impact and restore the device. The brushless motor is used for electrical energy generation. Note: The geometric dimensions and materials of the ASA device are listed in the **tables S3-S5**.





**Fig. S3. Dimensions of the ASA device.** *l* represents the overall length of the device, *w* signifies the overall width of the device, *h*_1_ is the height of the device’s outer frame, *h*_2_ is the maximum height of the device, and *h*_3_ is the travel distance of the sleeve.


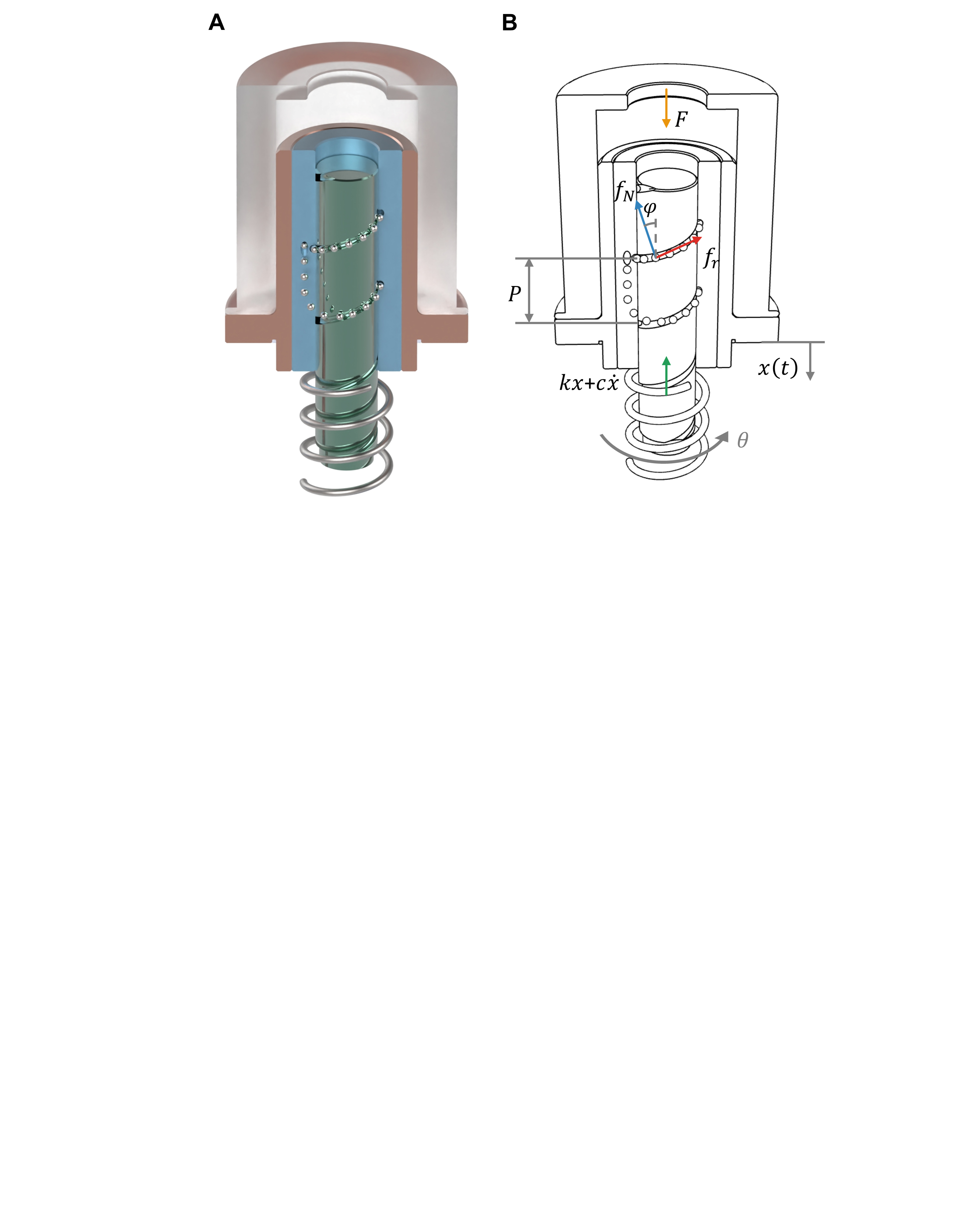


**Fig. S4. Mechanical simplified model of the sleeve and ball screw.**


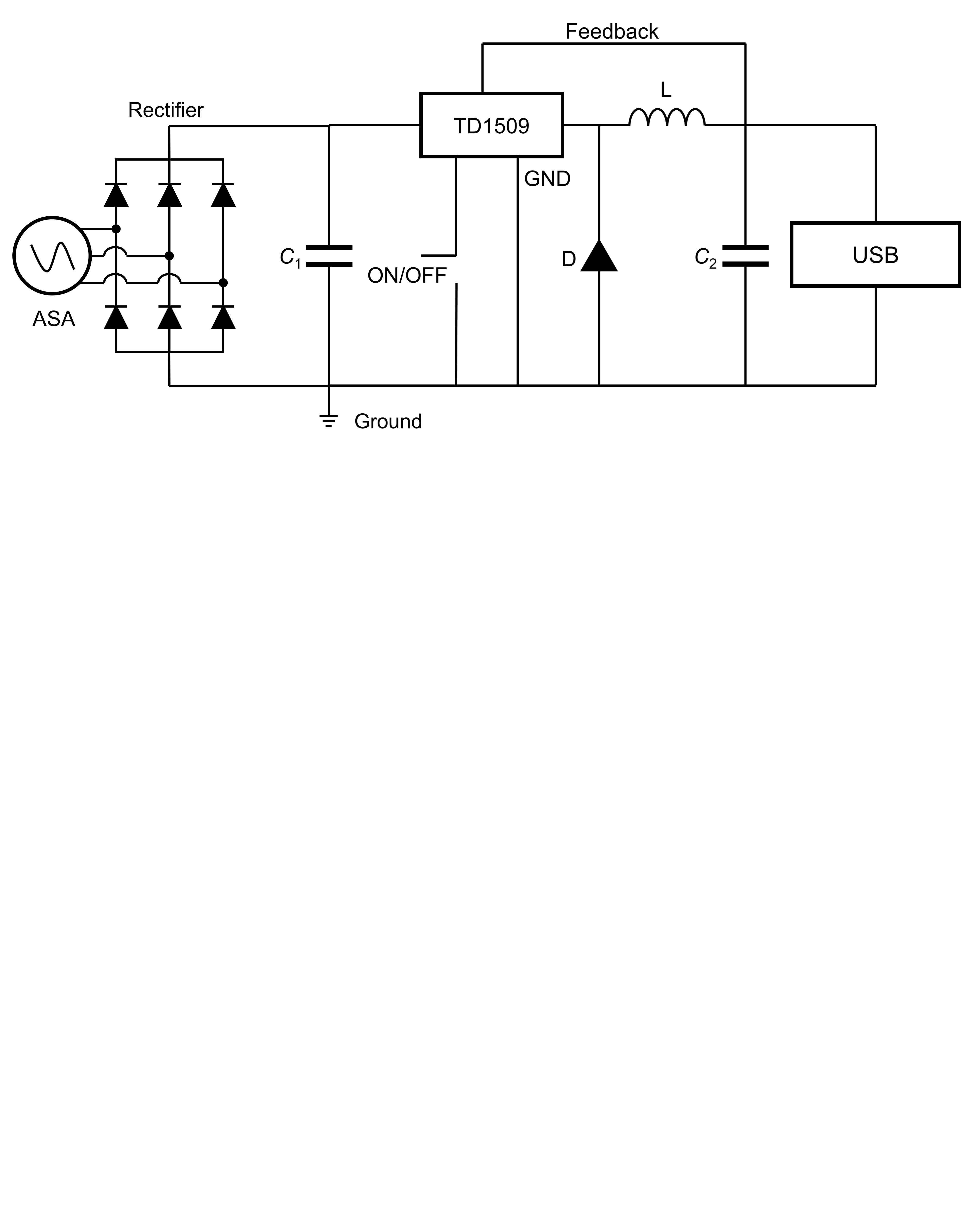


**Fig. S5. Power management circuit.**


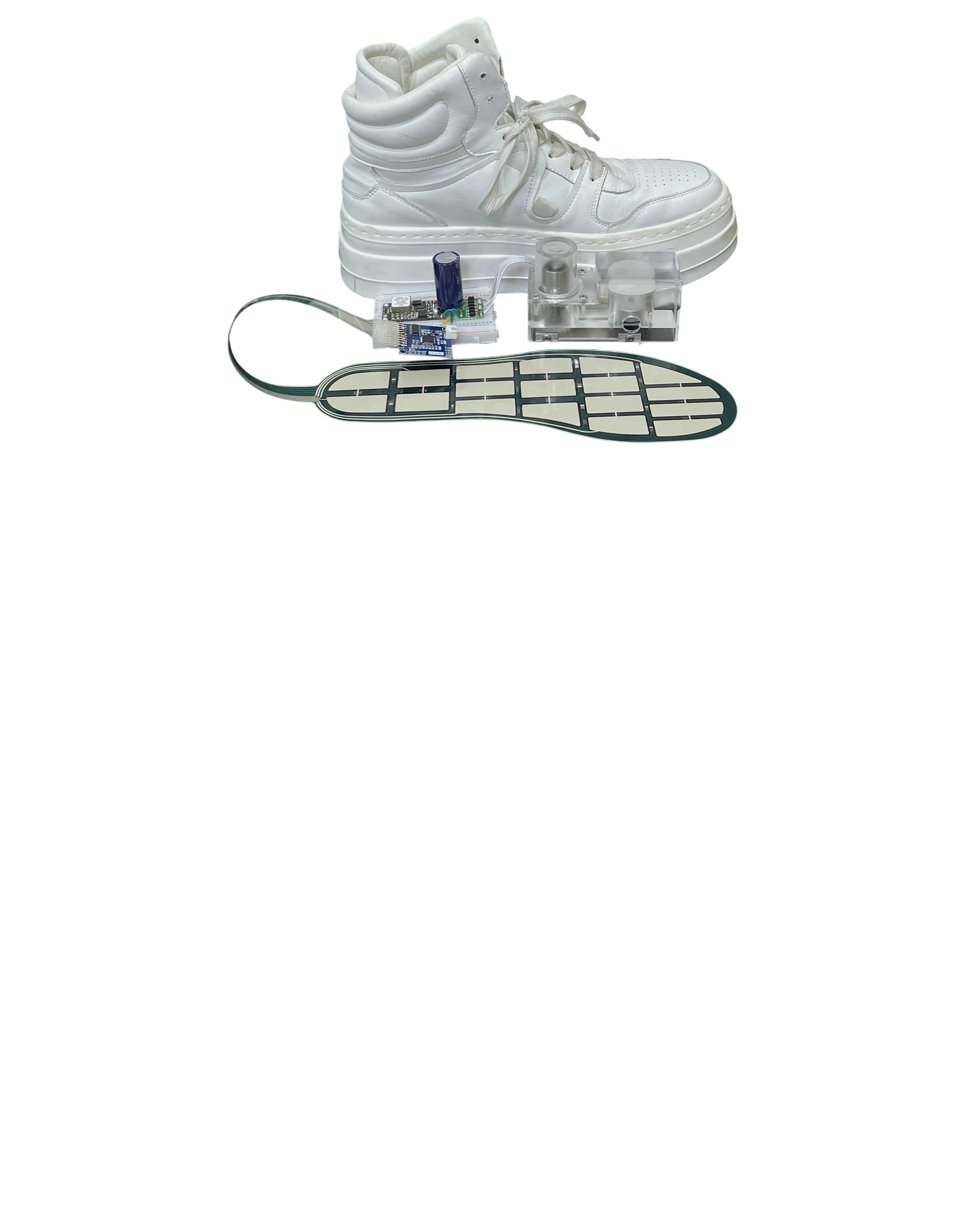


**Fig. S6. Fabricated prototype and associated components.**


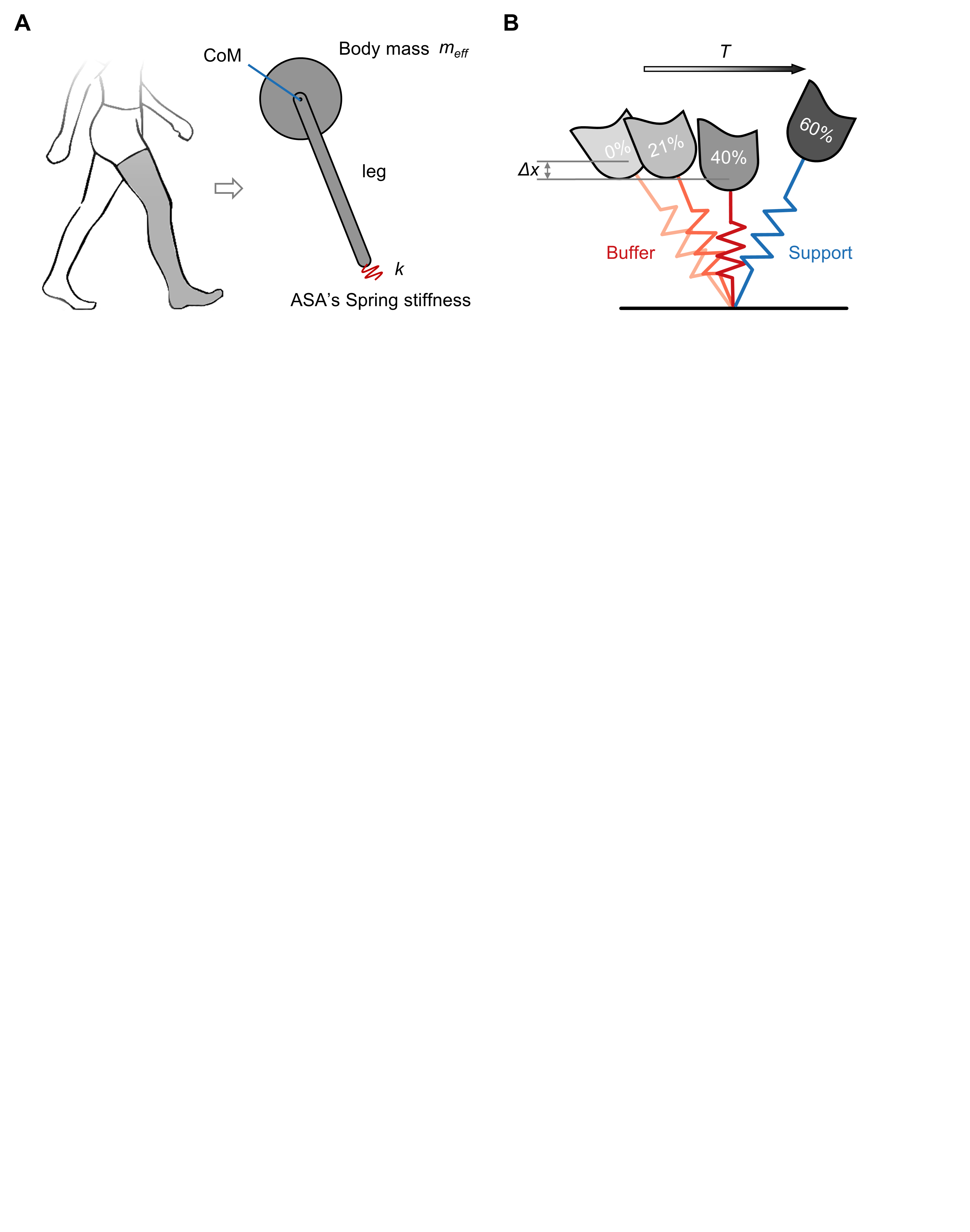


**Fig. S7.** **Simplified biomechanical model of the ASA device.** (**A**) The human body wearing the ASA is simplified into an inverted pendulum and a spring. (**B**) Biodynamics of the ASA at the moment of landing. The spring connected to the guide disc is compressed in the early stage of support, and the compression amount of the spring determines the vertical stroke (Δ*x*) of the sleeve. The relationship between the spring stiffness (*k*), the stroke of the sleeve (Δ*x*), and the effective body mass (*m_eff_*) is as described in **text S1**.


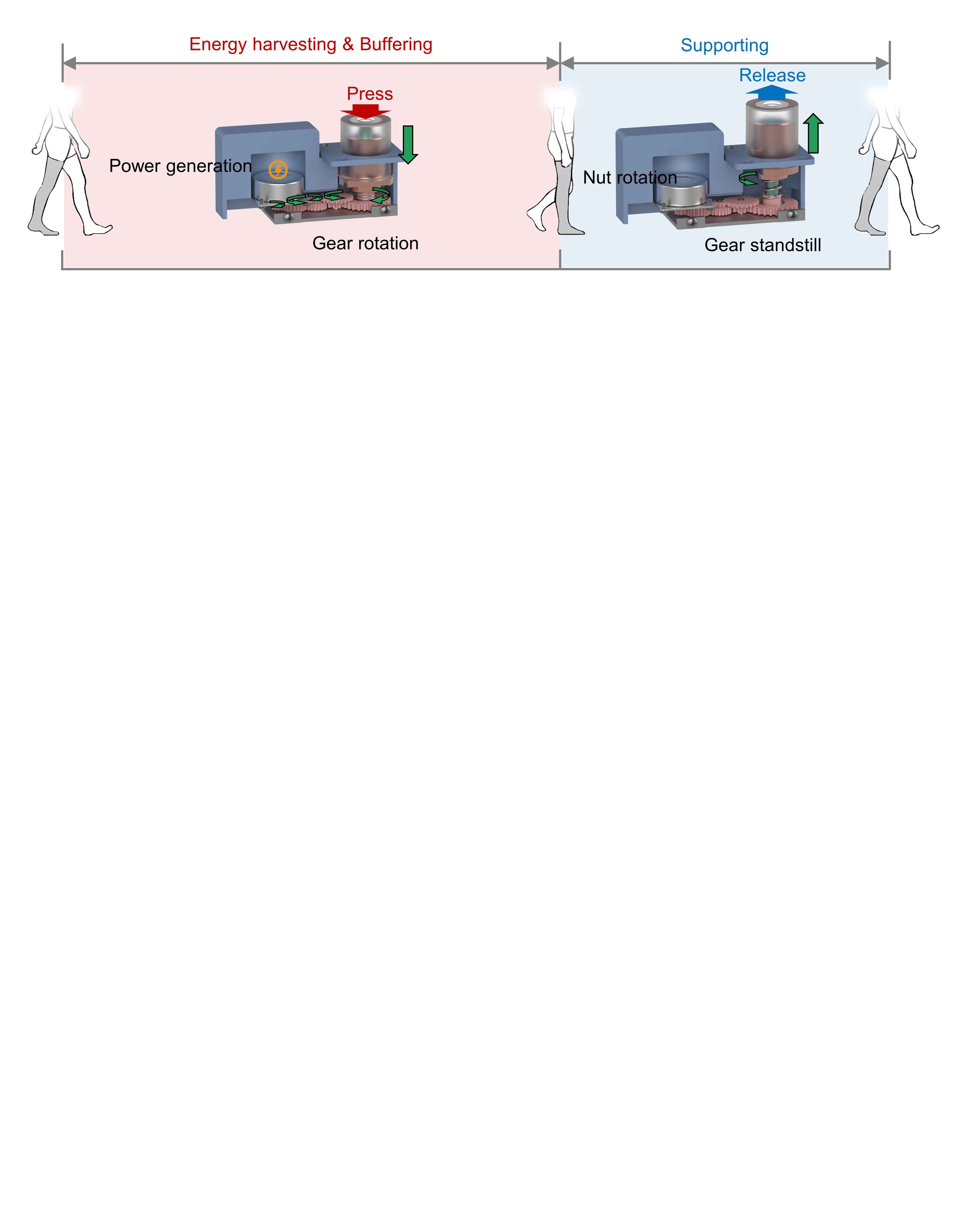


**Fig. S8. Mechanical working principle of the ASA device.** During the stance phase of the gait cycle, the ASA converts the energy from foot-ground impact into electrical energy. When the user’s arch presses against the ground, the spring in the device compresses, absorbing the impact energy while providing support to the arch. Simultaneously, the outer sleeve moves downward, driving the ball screw transmission mechanism, which converts linear motion into rotational motion. The gearbox amplifies the rotational frequency, ultimately driving the motor to rotate rapidly and generate electricity. When the body transitions to the swing phase, the gearbox prevents reverse rotation. The spring provides an upward thrust to the ball screw housing, which rotates relative to its outer sleeve and pushes the outer sleeve upward. This action provides a driving force to the arch, aiding in lifting the foot off the ground and restoring it to its original state.


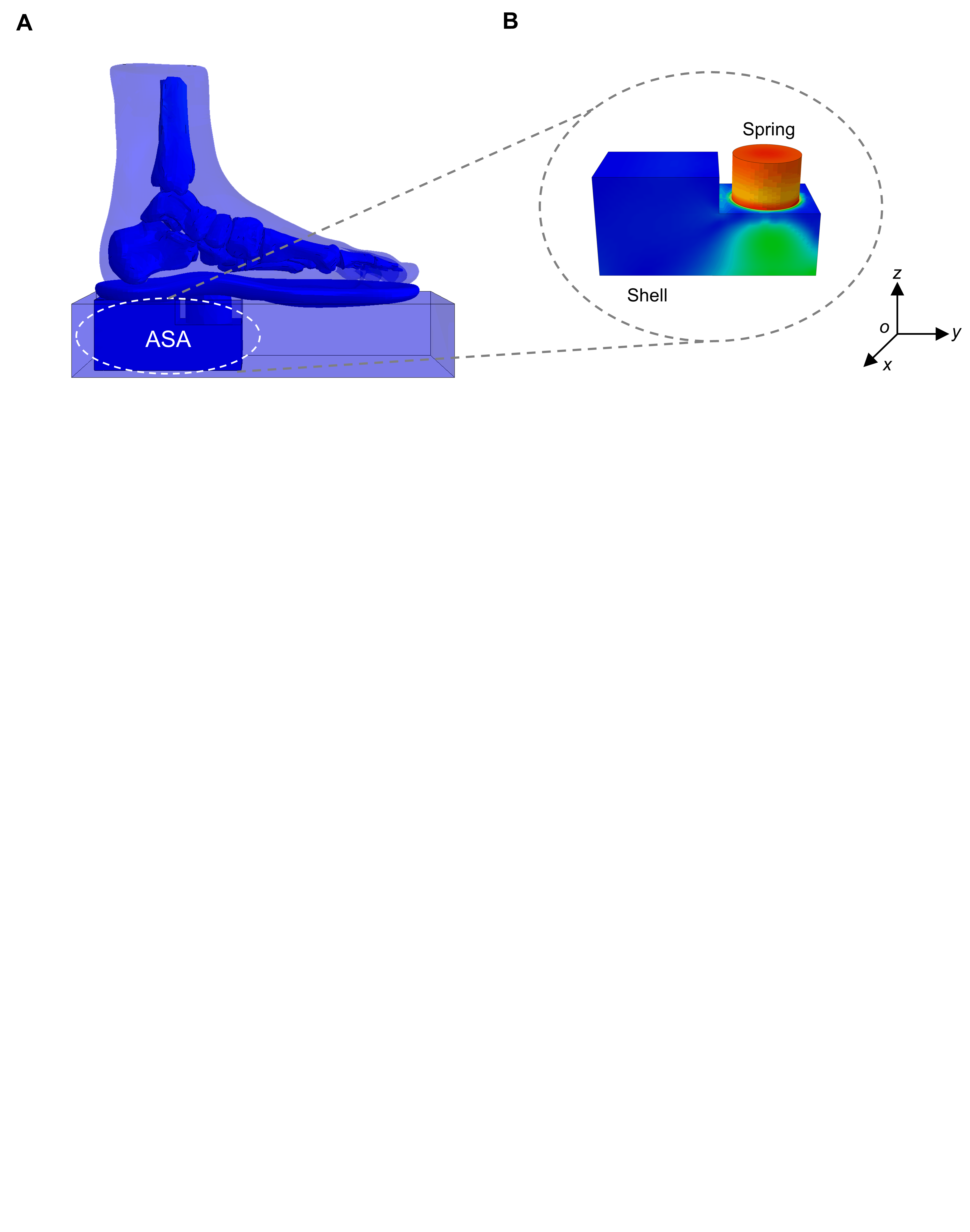


**Fig. S9. Simulated musculoskeletal model and ASA model.** (**A**) Musculoskeletal model wearing the ASA device. (**B**) ASA device model. The models are solved using ABAQUS SIMULIA software.


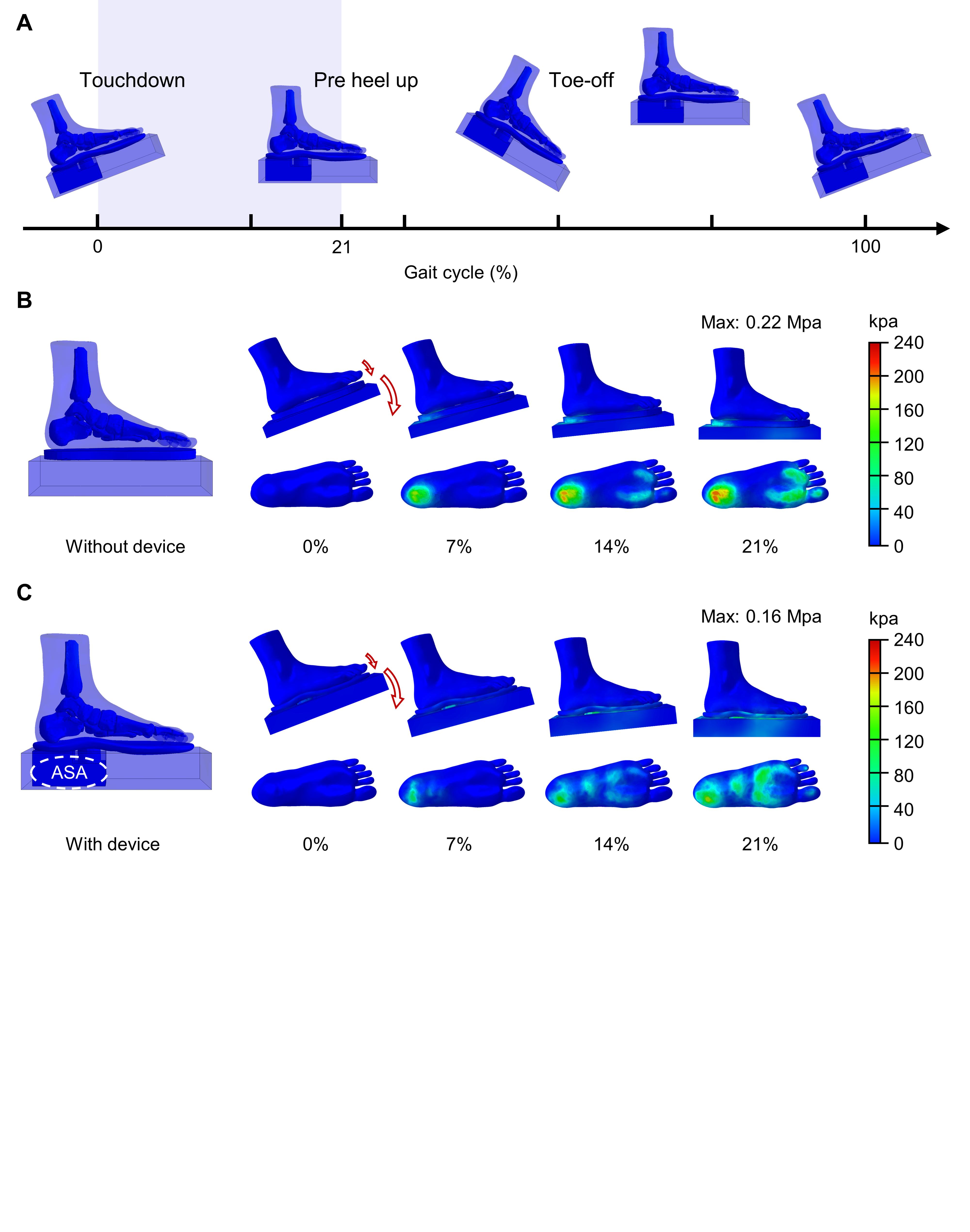


**Fig. S10. Dynamic characteristics of the planta.** (**A**) Foot landing cycle throughout the gait cycle. (**B**) Stress distribution of plantar pressure during landing without wearing the device. (**C**) Stress distribution of plantar pressure during landing with wearing the device. There is a small relative displacement between the plantar surface and the sole. The entire stride lasts for one second, and the stance phase accounts for approximately 21% of the whole gait cycle.


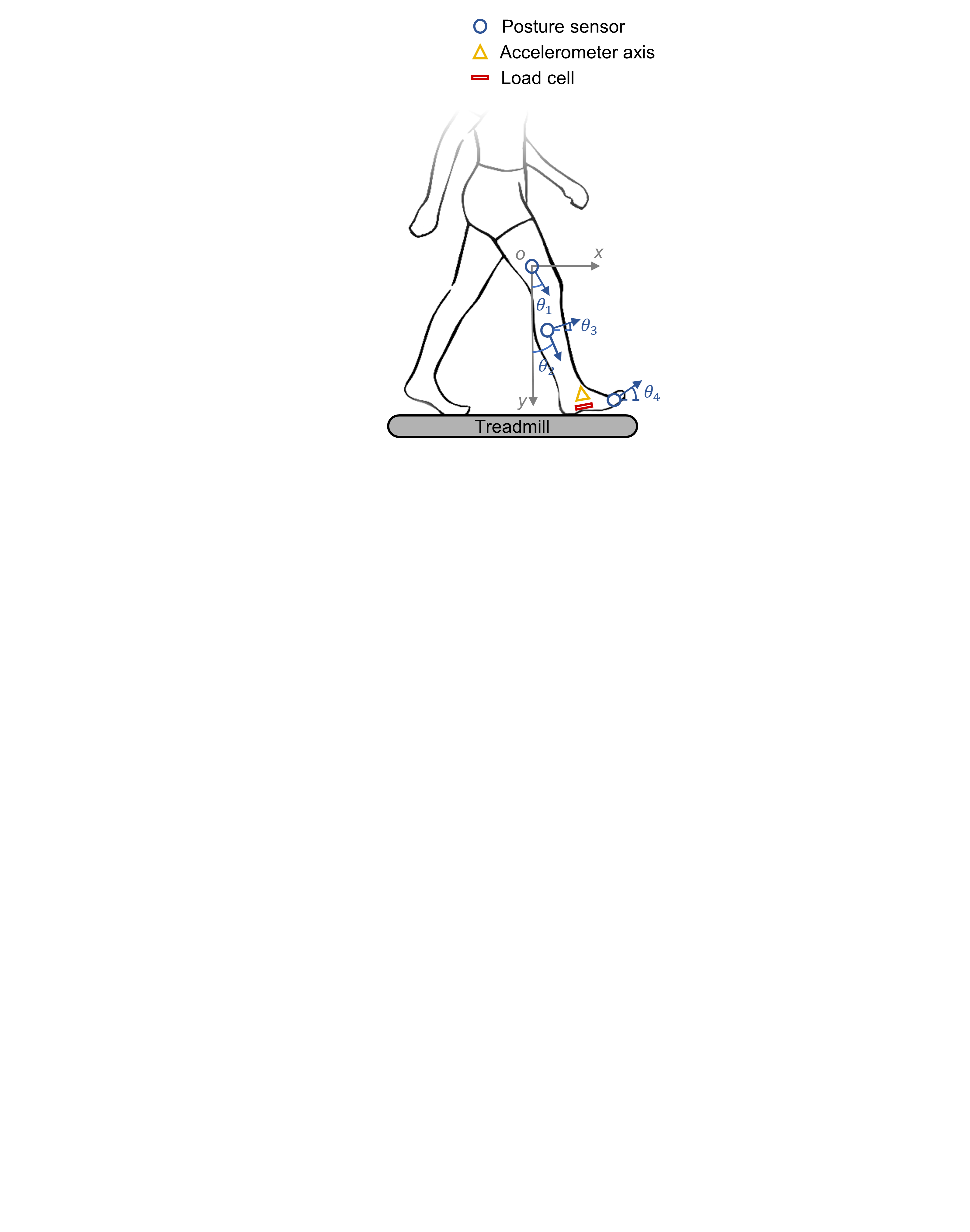


**Fig. S11. Wearing positions of the characterization device.** Six-axis attitude sensors are installed on the side of the thigh and calf, and the front side of the foot, respectively. An accelerometer is installed on the side of the arch. A load sensor is installed on the bottom of the arch.


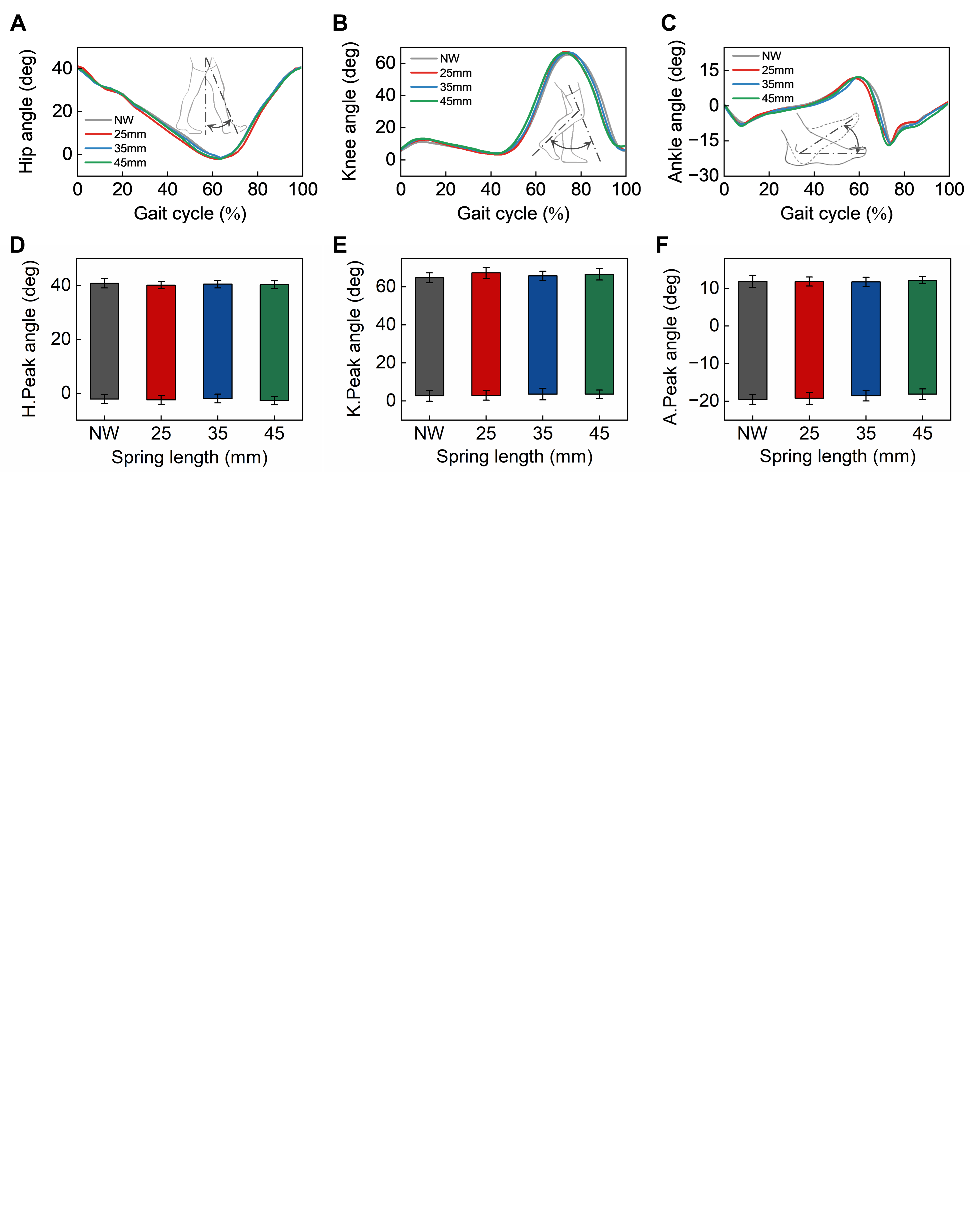


**Fig. S12. Angle variations in joint motions of hip, knee, and ankle.** (**A**) Angle variations of the hip joint in a gait cycle when wearing the ASA device equipped with springs of different stiffnesses. (**B**) Angle variations of the knee joint in a gait cycle when wearing the ASA device equipped with springs of different stiffnesses. (**C**) Angle variations of the ankle joint in a gait cycle when wearing the ASA device equipped with springs of different stiffnesses. (**D**) Comparison of the peak angle of the hip joint in a gait cycle with and without wearing the ASA device equipped with springs of different stiffnesses. (**E**) Comparison of the peak angle of the knee joint in a gait cycle with and without wearing the ASA device equipped with springs of different stiffnesses. (**F**) Comparison of the peak angle of the ankle joint in a gait cycle with and without wearing the ASA device equipped with springs of different stiffnesses. There is no significant difference in the angles of the hip, ankle, and knee joints when walking with the ASA device equipped with springs of different stiffnesses.


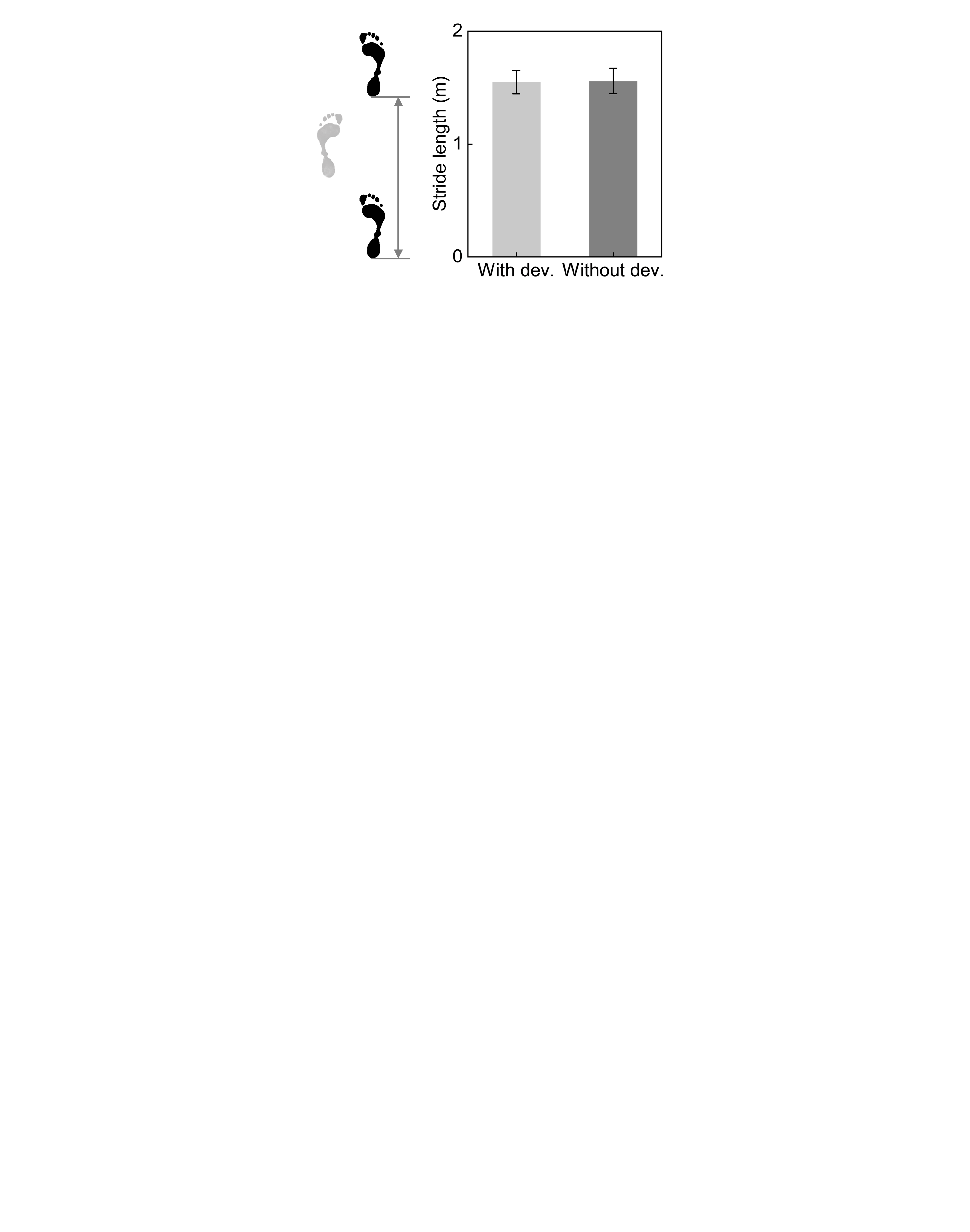


**Fig. S13. Distance of stride length with and without device (dev.).** There is no significant difference in stride length when walking with and without the ASA device.





**Fig. S14. Open-circuit voltage of the energy harvesting component under six types of gait dynamics.** The output voltage fluctuation of the ASA is attributed to the highly integrated characteristics of human walking, which is consistent with the gait characteristics during human walking and the dynamic differences between gait patterns.


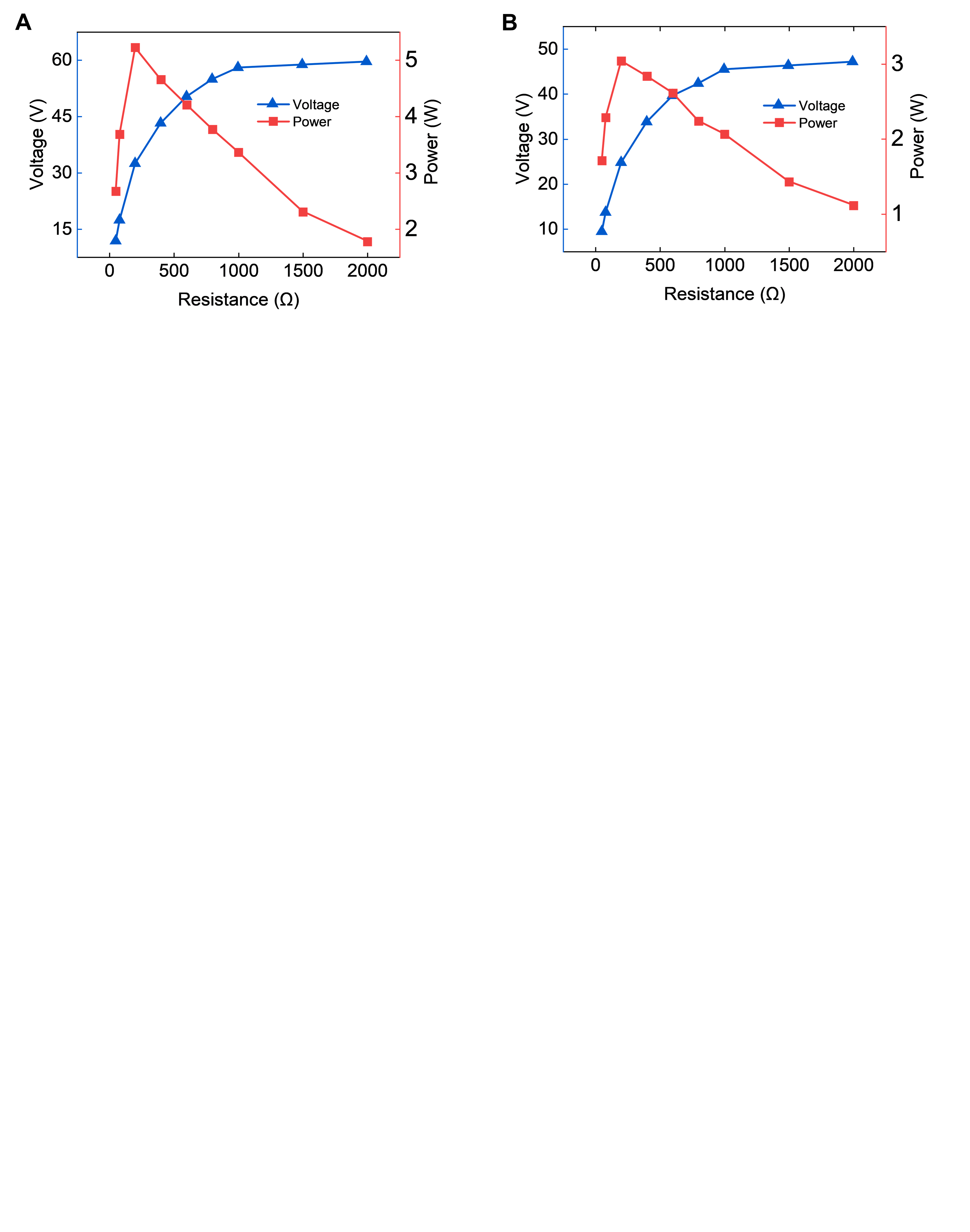


**Fig. S15. Power output and voltage output of the ASA when the external resistance changes.** (**A**) Power output and voltage output before rectification. (**B**) Power output and voltage output after rectification.


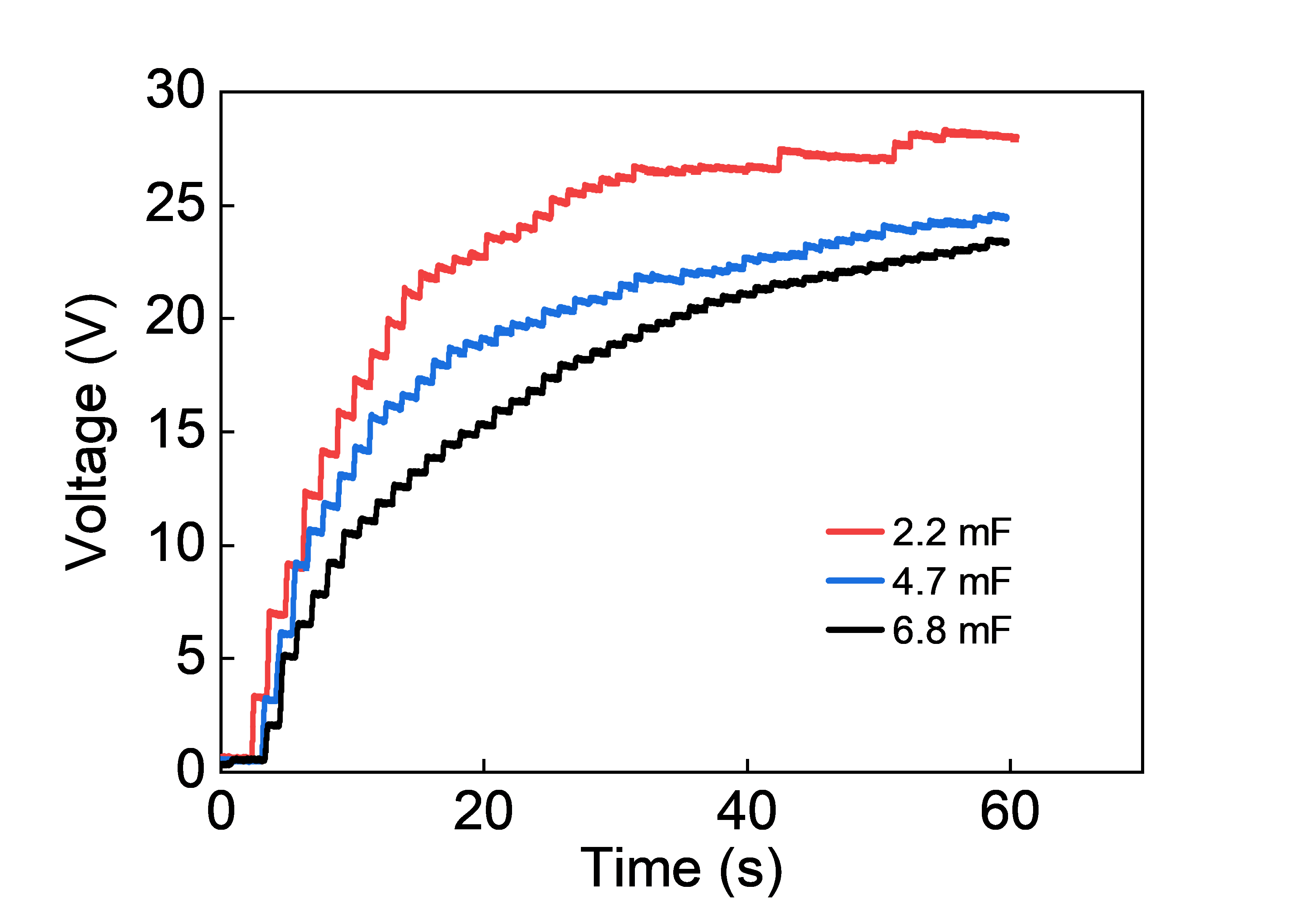


**Fig. S16. Charging curves of 2.2 mF, 4.7 mF, and 8.2 mF capacitors.**

**
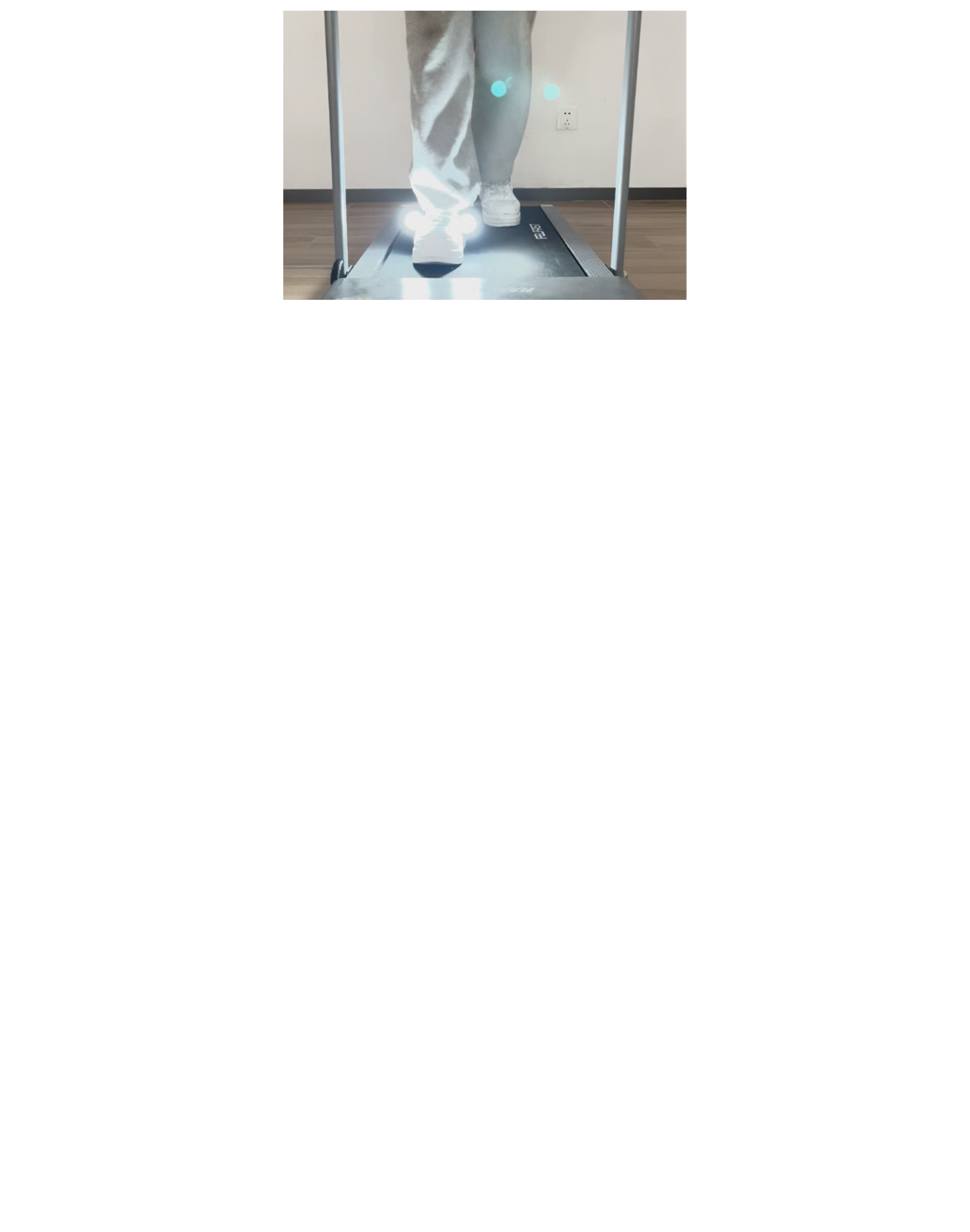
**

**Fig. S17. Lighting up two 3 W diode bulbs.** The power output generated at a walking speed of 1.5 m/s can light up two 3 W light-emitting diode bulbs simultaneously.

**
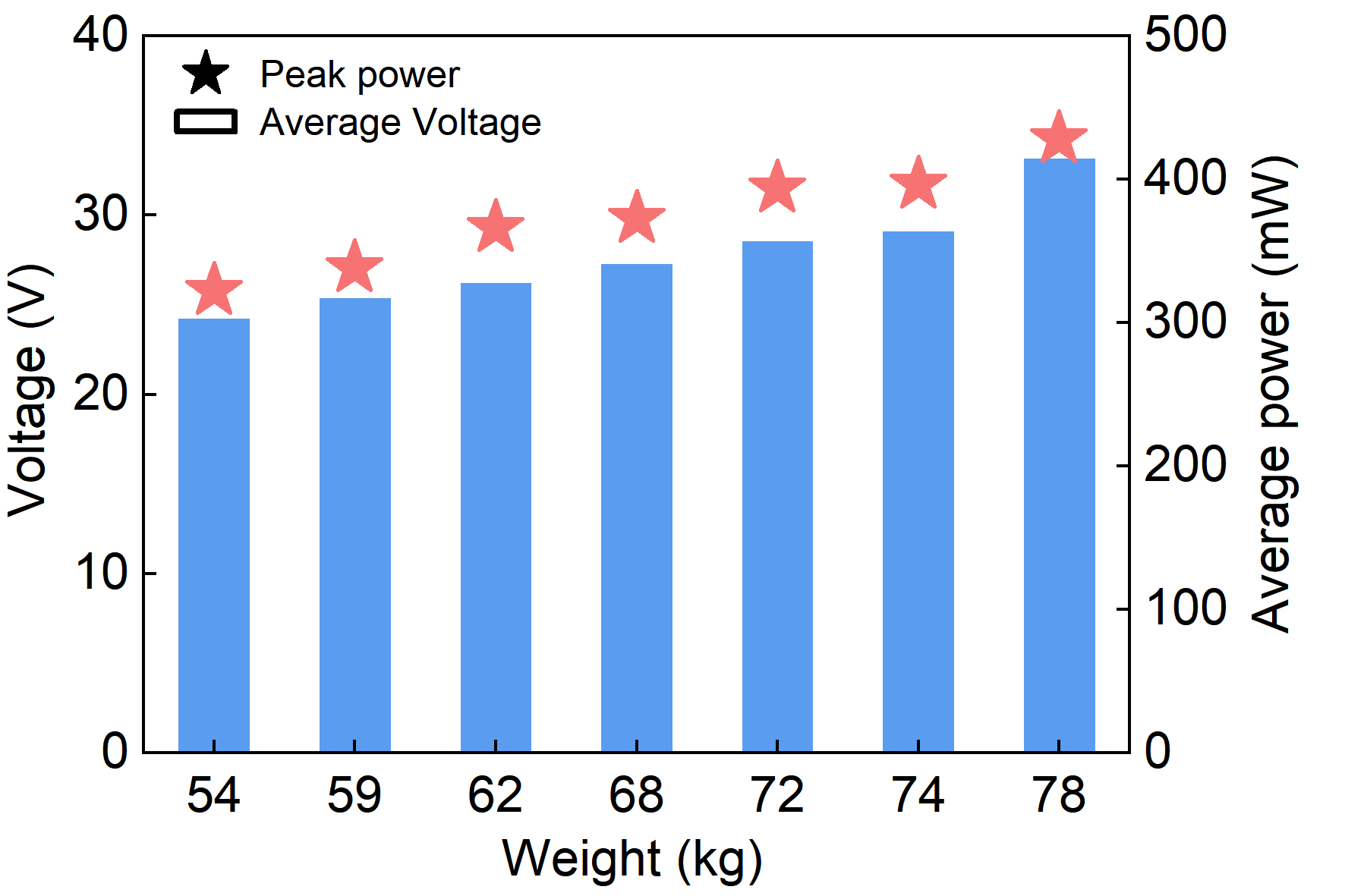
**

**Fig. S18. The output voltage and average power of seven experimental subjects with different weights.** To address the inter-individual variability, we analyze the energy harvesting performance across participants with different body weights (ranging from 54 to 78 kg). As illustrated, the output voltage and average power exhibit a positive correlation with body mass, attributed to the greater mechanical impact forces generated by heavier individuals. Importantly, even for the participant with the lowest body weight (54 kg), the generated power remains sufficient to drive the system, confirming the FS-DWS’s universality and robustness across a diverse user population.


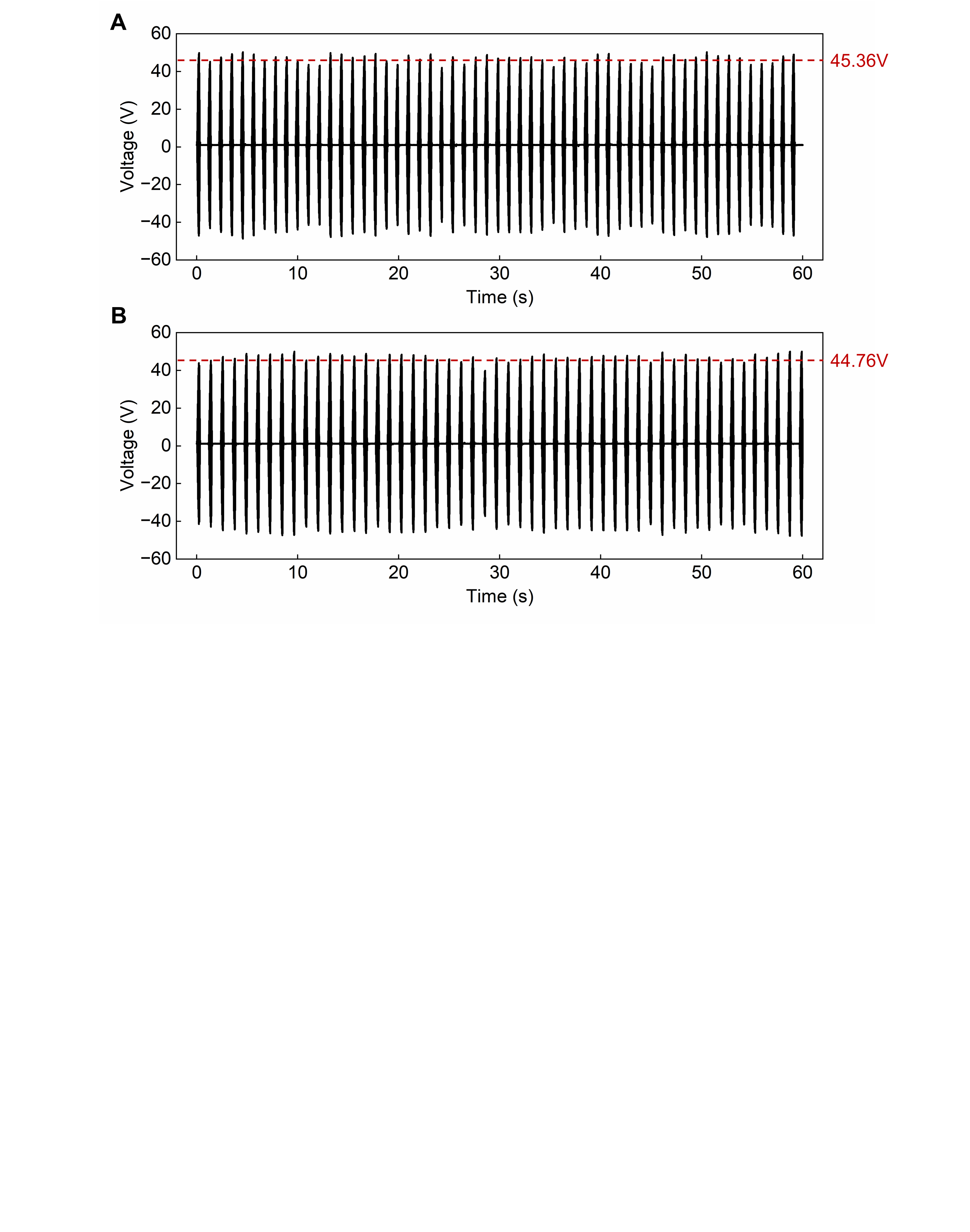


**Fig. S19. Output voltage of the ASA device.** (**A**) Initial measurement. (**B**) Measurement after 9 months.


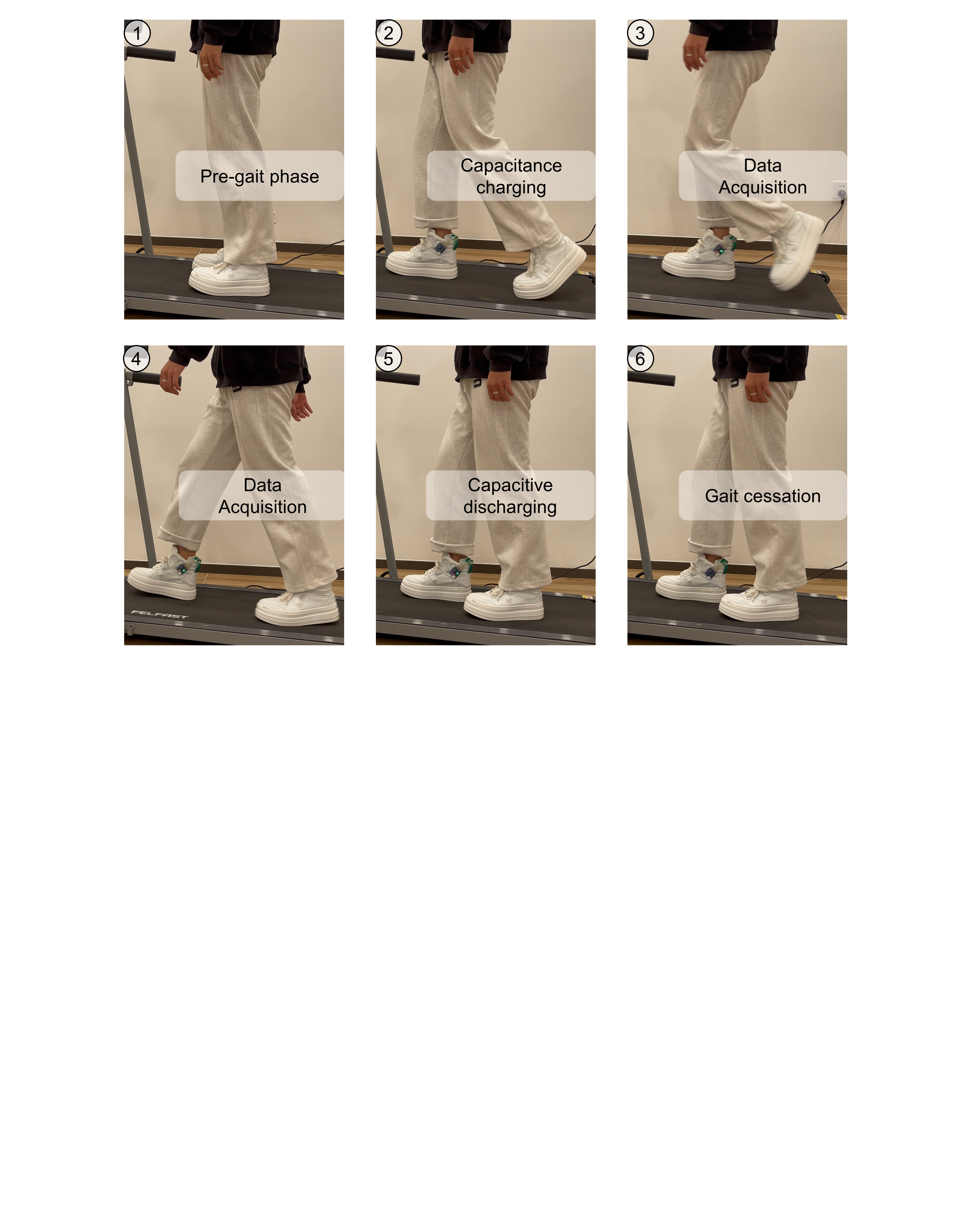


**Fig. S20.** **Fully self-powered process of the wearable device system.** When a tester starts walking, the green light turns on, indicating that the wearable sensing system is in the charging phase; during human walking, both the green light and the red light are on, indicating that the wearable sensing system is operating normally and is synchronously transmitting plantar pressure data to the client; immediately after the tester stops walking, the wearable sensing system is consuming the remaining electrical energy generated during walking; after a period of time following the end of walking, the wearable sensing system shuts down after consuming all the remaining electrical energy.

**Note:** As shown in movie S4, when starting to walk, the WSS system needs to be activated for the first time, and it takes 5 seconds to charge. When the WSS system starts to operate, the system latency is less than 100 ms, which is so low that it is almost imperceptible to the human eye, and it is sufficient to meet the real-time requirements.


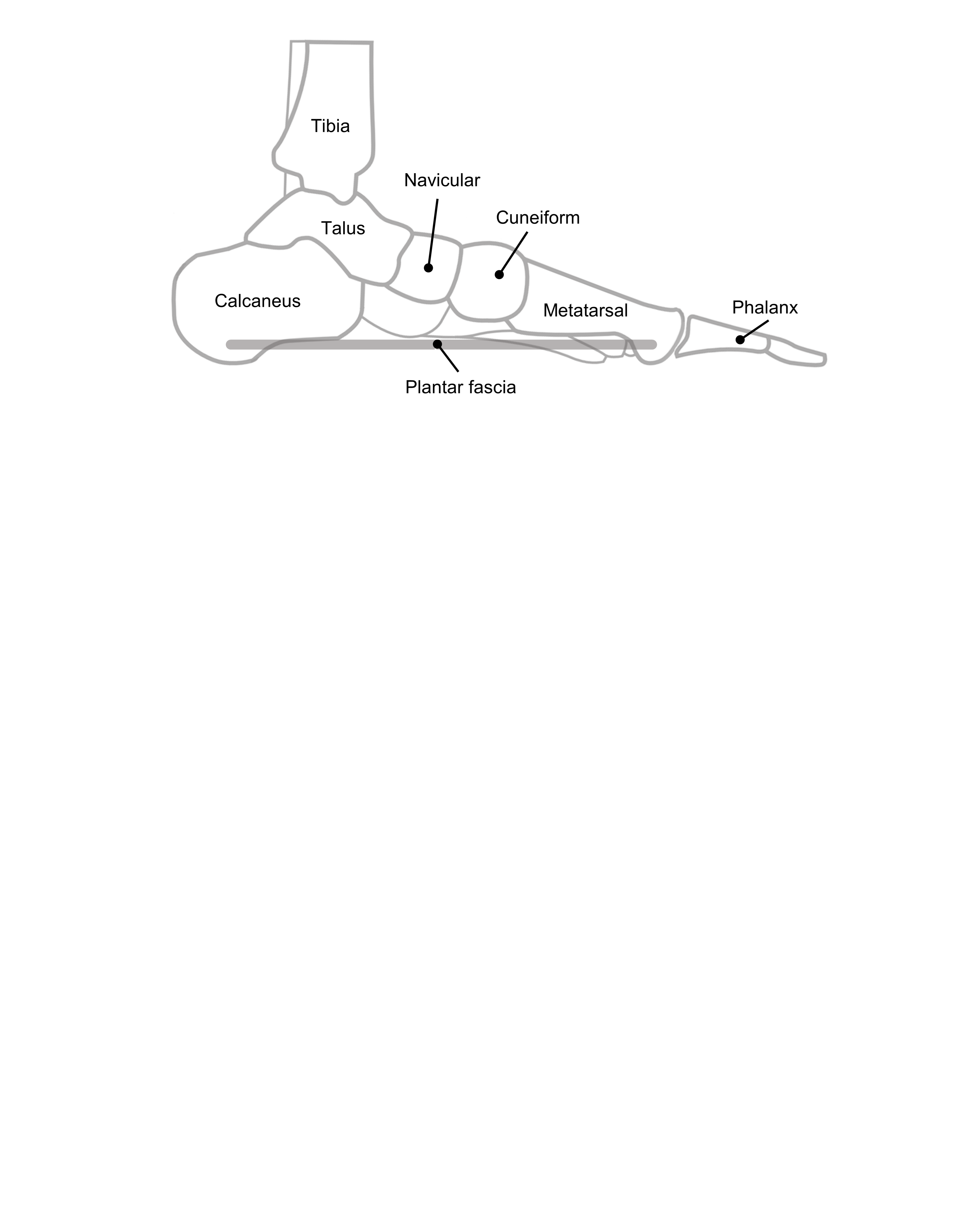


**Fig. S21. Composition of the human foot bones.**





**Fig. S22. The thermal performance of the WSS.** (A) The thermal performance at 0 min. (B) The thermal performance at 1 min. (C) The thermal performance at 5 min. (D) The thermal performance at 10 min. A high-resolution infrared thermal imager is used to monitor the surface temperature of the WSS while a subject walks on a treadmill at a constant speed (1.5 m/s) for 10 min. The data confirms that the internal power management circuit and Bluetooth module operate at high efficiency with minimal heat generation, ensuring user safety and comfort.


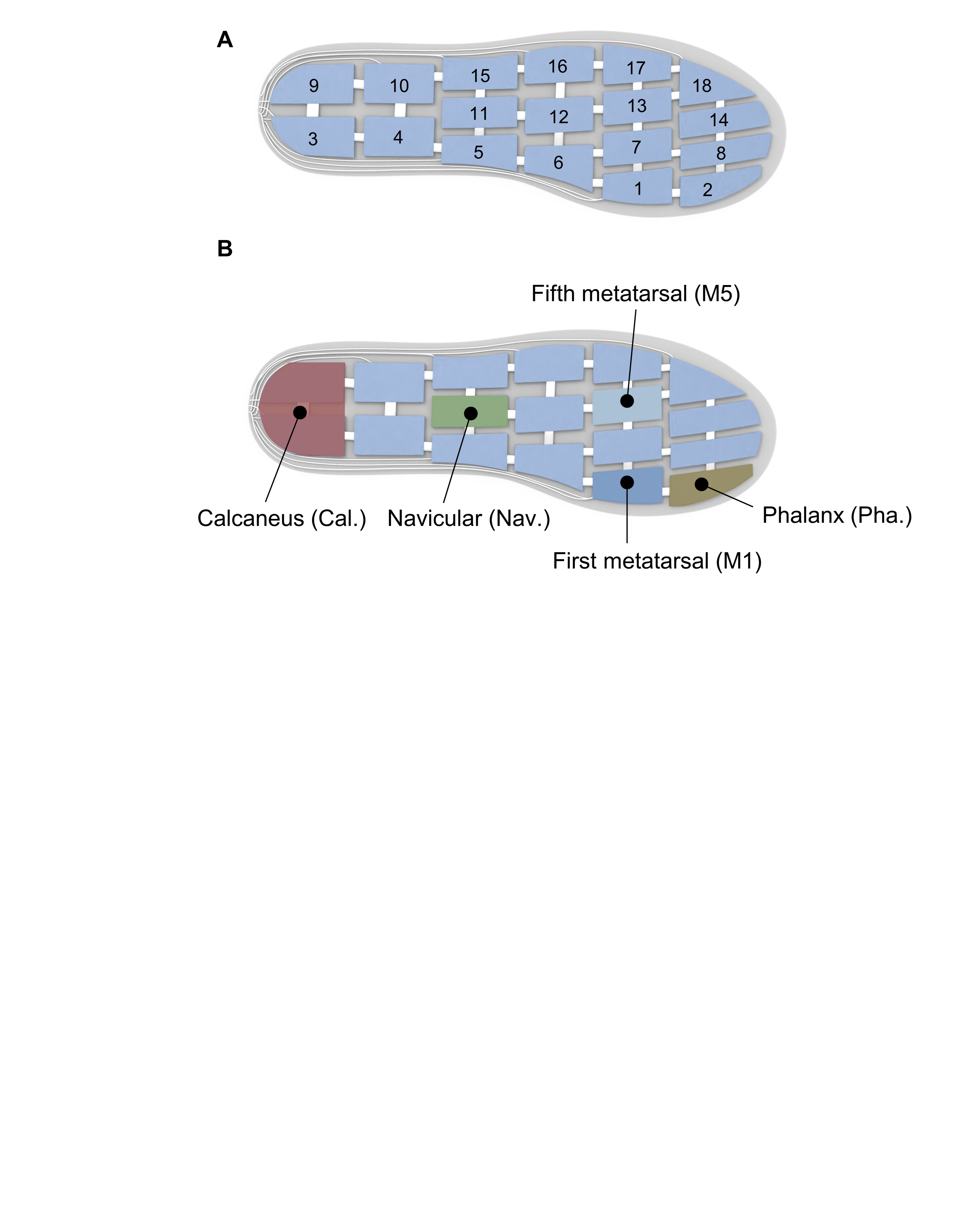


**Fig. S23. Arrangement of the 18 pressure sensing units in the flexible sensor array.** Units 3 and 9 reflect pressure in the heel region, unit 11 reflects pressure in the navicular region, unit 1 reflects pressure in the first metatarsal region, unit 13 reflects pressure in the fifth metatarsal region, and unit 2 reflects pressure in the phalanx region.

**Note:** The flexible sensing insole utilizes a spatially distributed flexible sensor array rather than single-point sensors. These large-area sensing units form ‘sensing zones’ that cover key anatomical landmarks (e.g., calcaneus, metatarsals), ensuring effective pressure data acquisition despite minor variations in foot placement or size. Furthermore, the ASA device features a compact, modular design embedded in the shoe heel, not sensitive to size. The device successfully provided consistent support and energy harvesting for all subjects. Future commercial iterations could further optimize this by offering graded module sizes (e.g., S/M/L) to accommodate extreme size ranges.


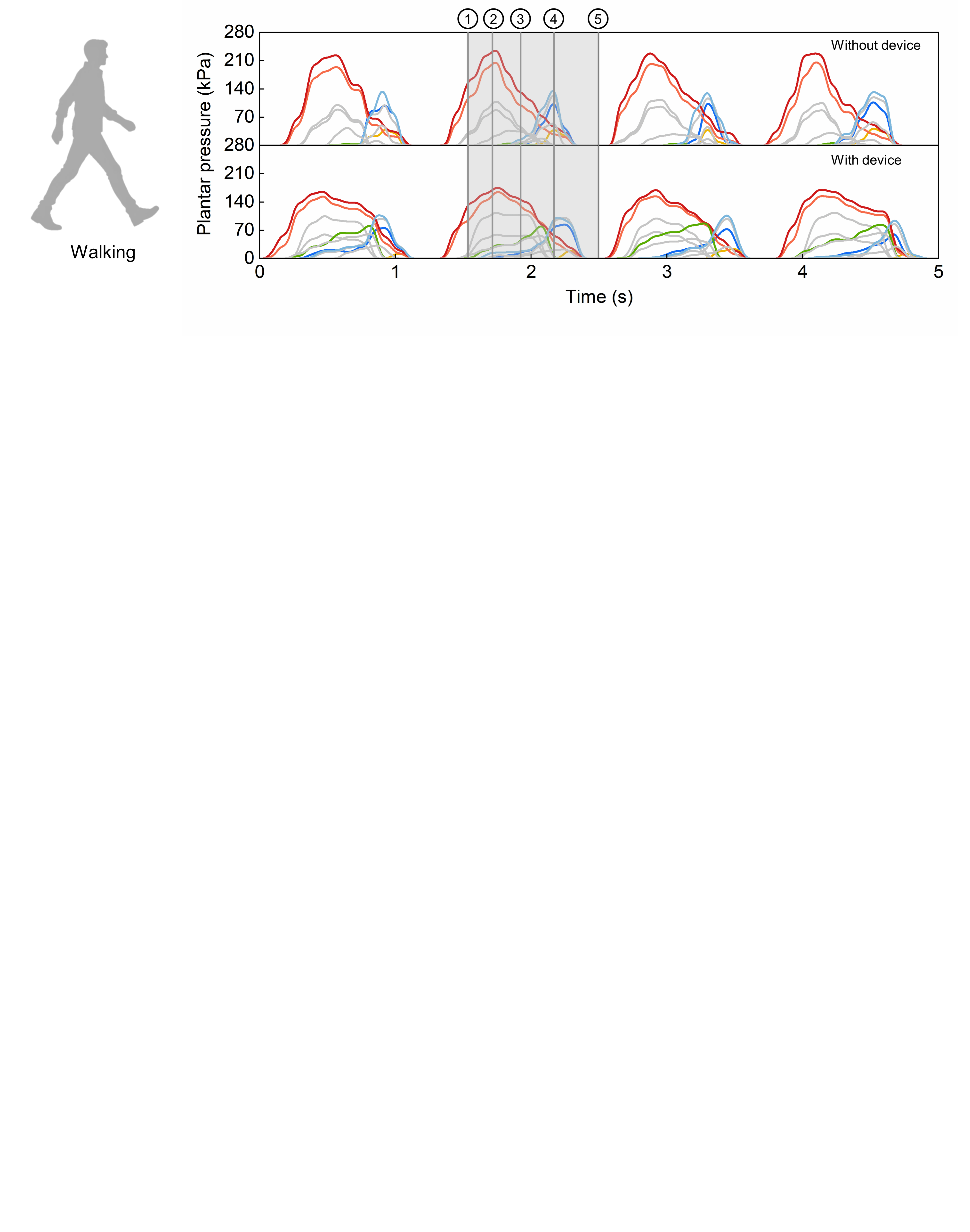


**Fig. S24.** **Plantar pressure data during walking when wearing and not wearing the device.** Four gait cycle situations of the foot sole pressure waveforms when walking with and without wearing the device, where the five gray vertical lines correspond to the typical sampling points in the pressure distribution map shown in **Fig. 3C**.


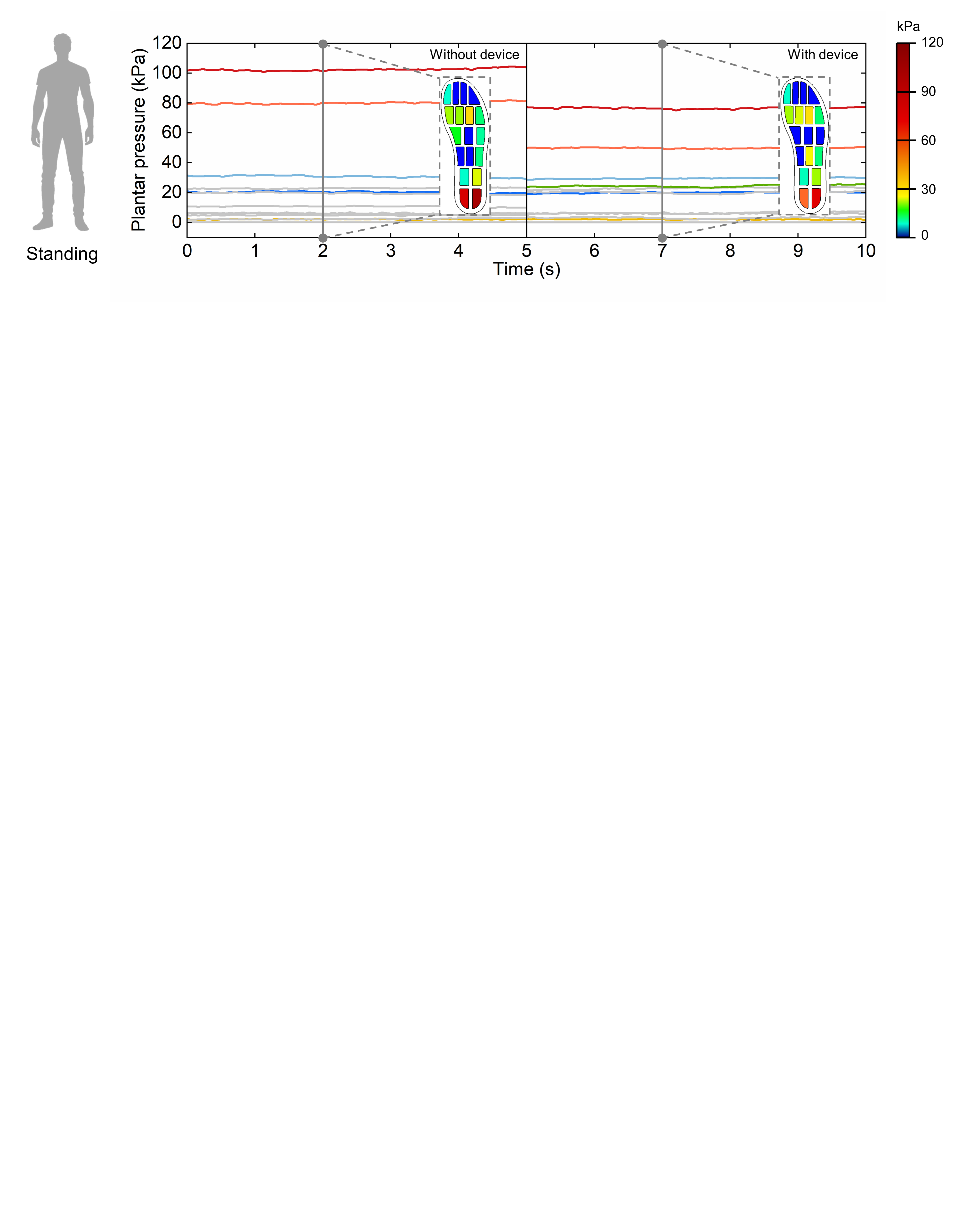


**Fig. S25. Pressure data curves and maps during standing when wearing and not wearing the device.**


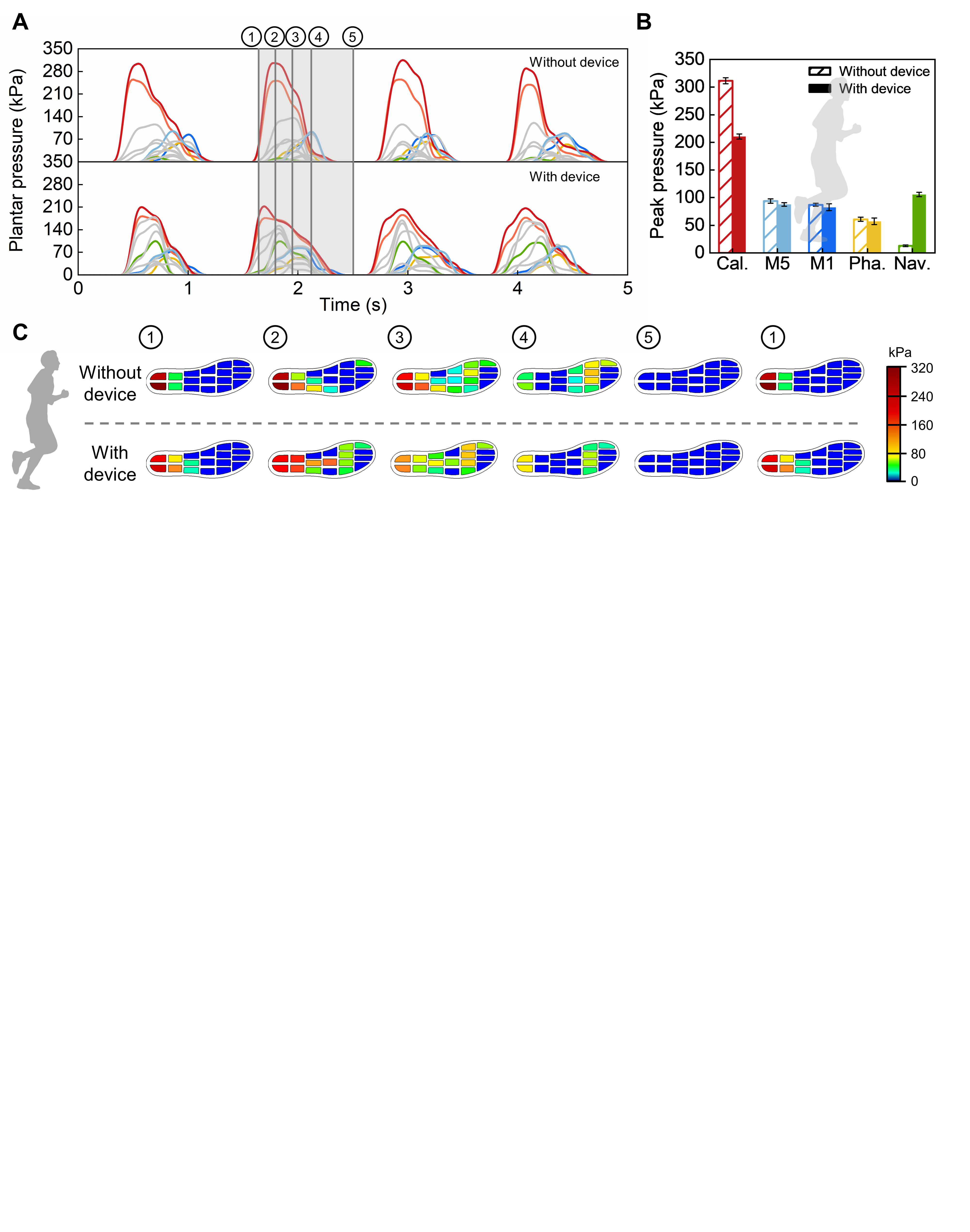


**Fig. S26. Pressure data curves and maps during jogging when wearing and not wearing the device.** (**A**) Pressure data curves in four gait cycles during jogging with and without the device. (**B**) Maximum peak pressure corresponding to Cal., M5, M1, Pha., and Nav. regions, respectively. (**C**) Dynamic pressure distribution corresponding to the typical sampling points of the gray vertical lines.


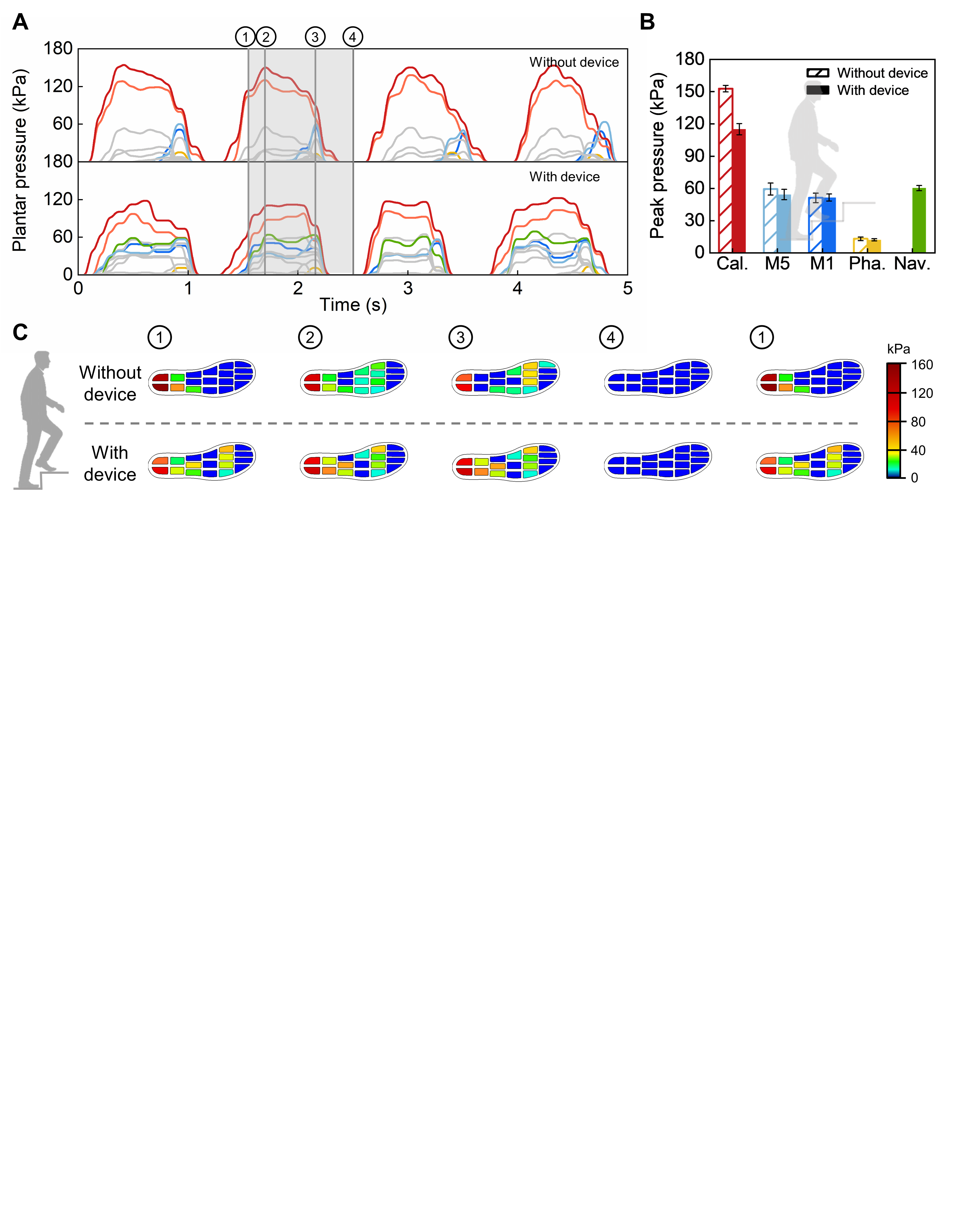


**Fig. S27. Pressure data curves and maps during going upstairs when wearing and not wearing the device.** (**A**) Pressure data curves in four gait cycles during going upstairs with and without the device. (**B**) Maximum peak pressure corresponding to Cal., M5, M1, Pha., and Nav. regions, respectively. (**C**) Dynamic pressure distribution corresponding to the typical sampling points of the gray vertical lines.


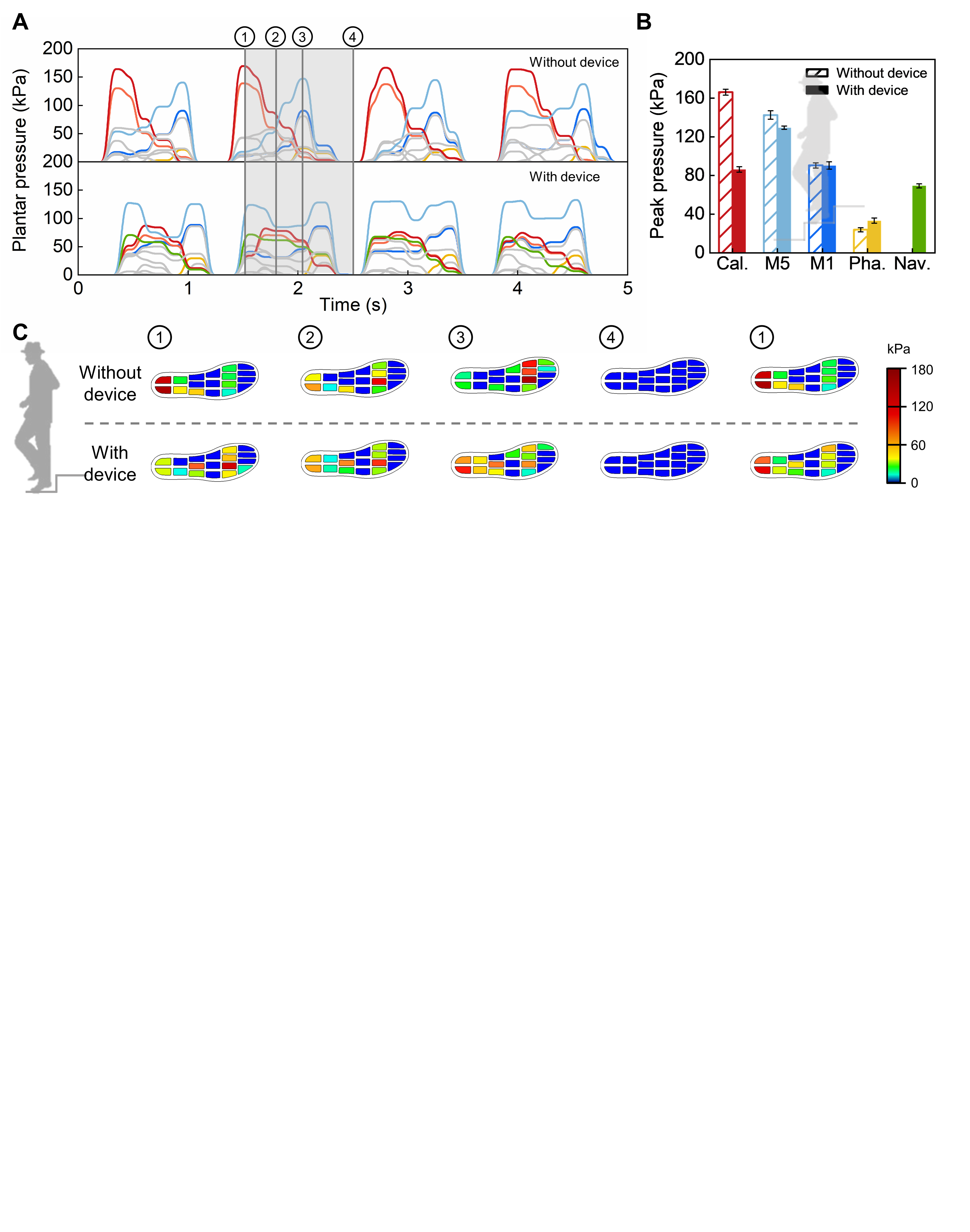


**Fig. S28. Pressure data curves and maps during going downstairs when wearing and not wearing the device.** (**A**) Pressure data curves in four gait cycles during going downstairs with and without the device. (**B**) Maximum peak pressure corresponding to Cal., M5, M1, Pha., and Nav. regions, respectively. (**C**) Dynamic pressure distribution corresponding to the typical sampling points of the gray vertical lines.


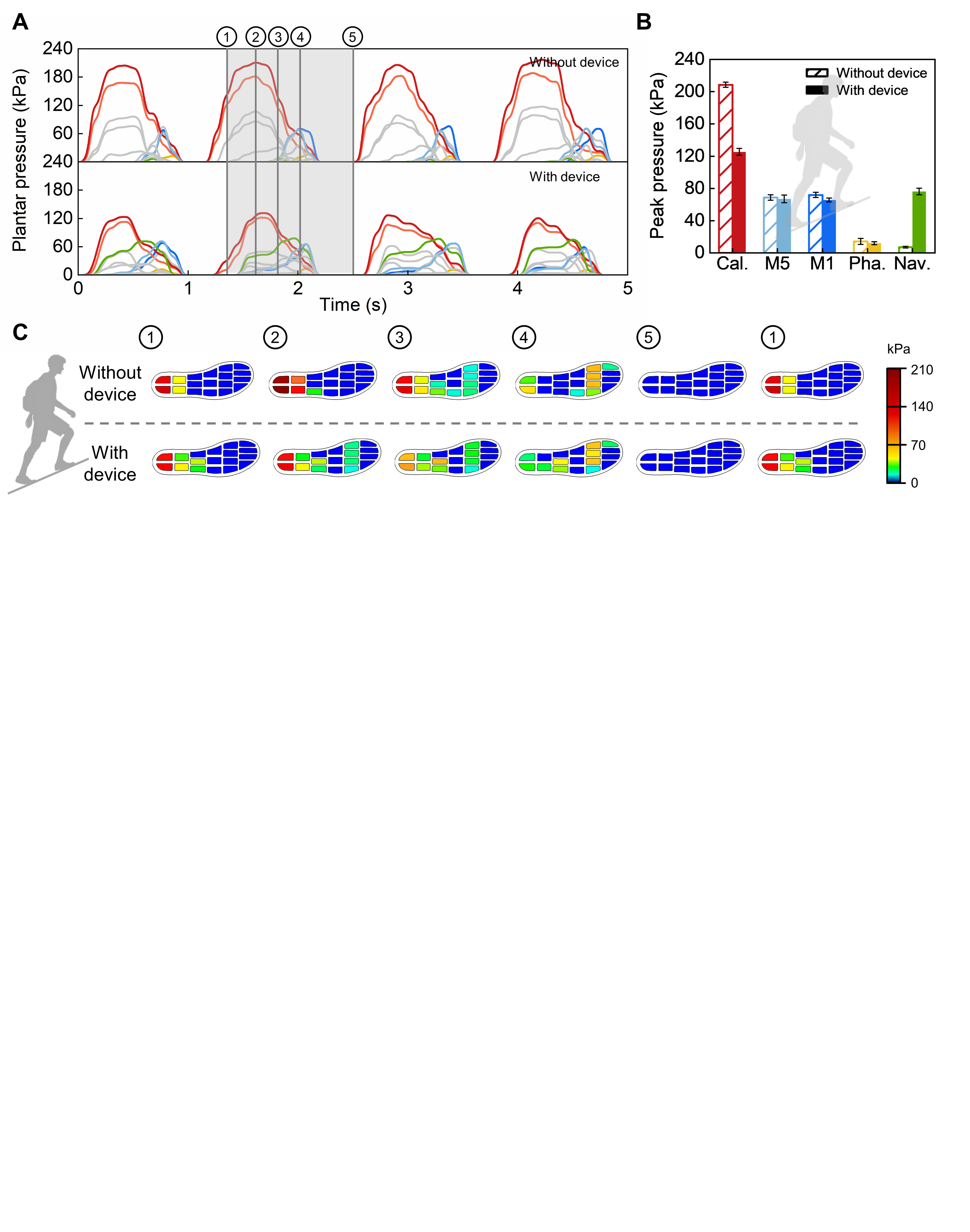


**Fig. S29. Pressure data curves and maps during going upslope when wearing and not wearing the device.** (**A**) Pressure data curves in four gait cycles during going upslope with and without the device. (**B**) Maximum peak pressure corresponding to Cal., M5, M1, Pha., and Nav. regions, respectively. (**C**) Dynamic pressure distribution corresponding to the typical sampling points of the gray vertical lines.


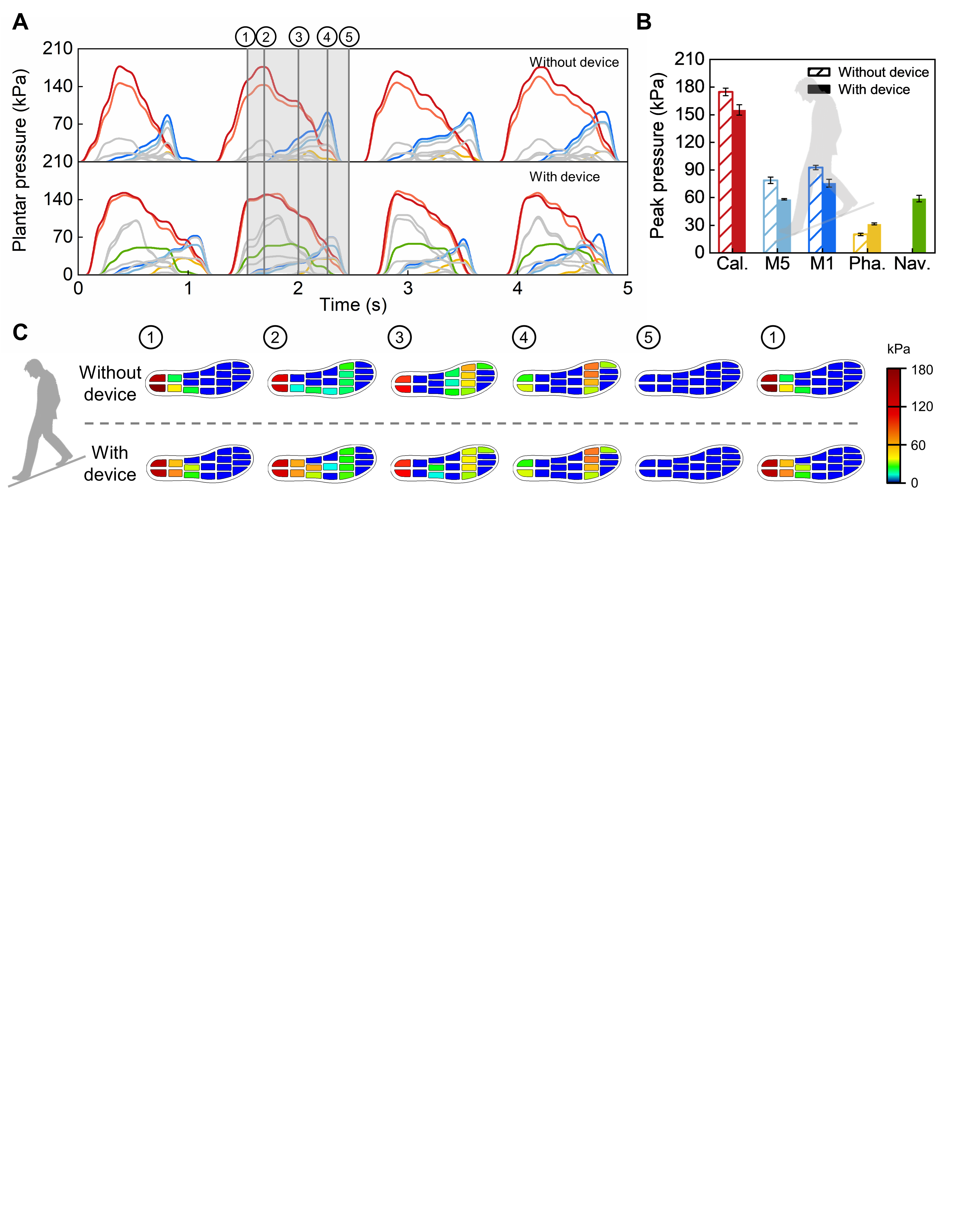


**Fig. S30. Pressure data curves and maps during going downslope when wearing and not wearing the device.** (**A**) Pressure data curves in four gait cycles during going downslope with and without the device. (**B**) Maximum peak pressure corresponding to Cal., M5, M1, Pha., and Nav. Regions, respectively. (**C**) Dynamic pressure distribution corresponding to the typical sampling points of the gray vertical lines.


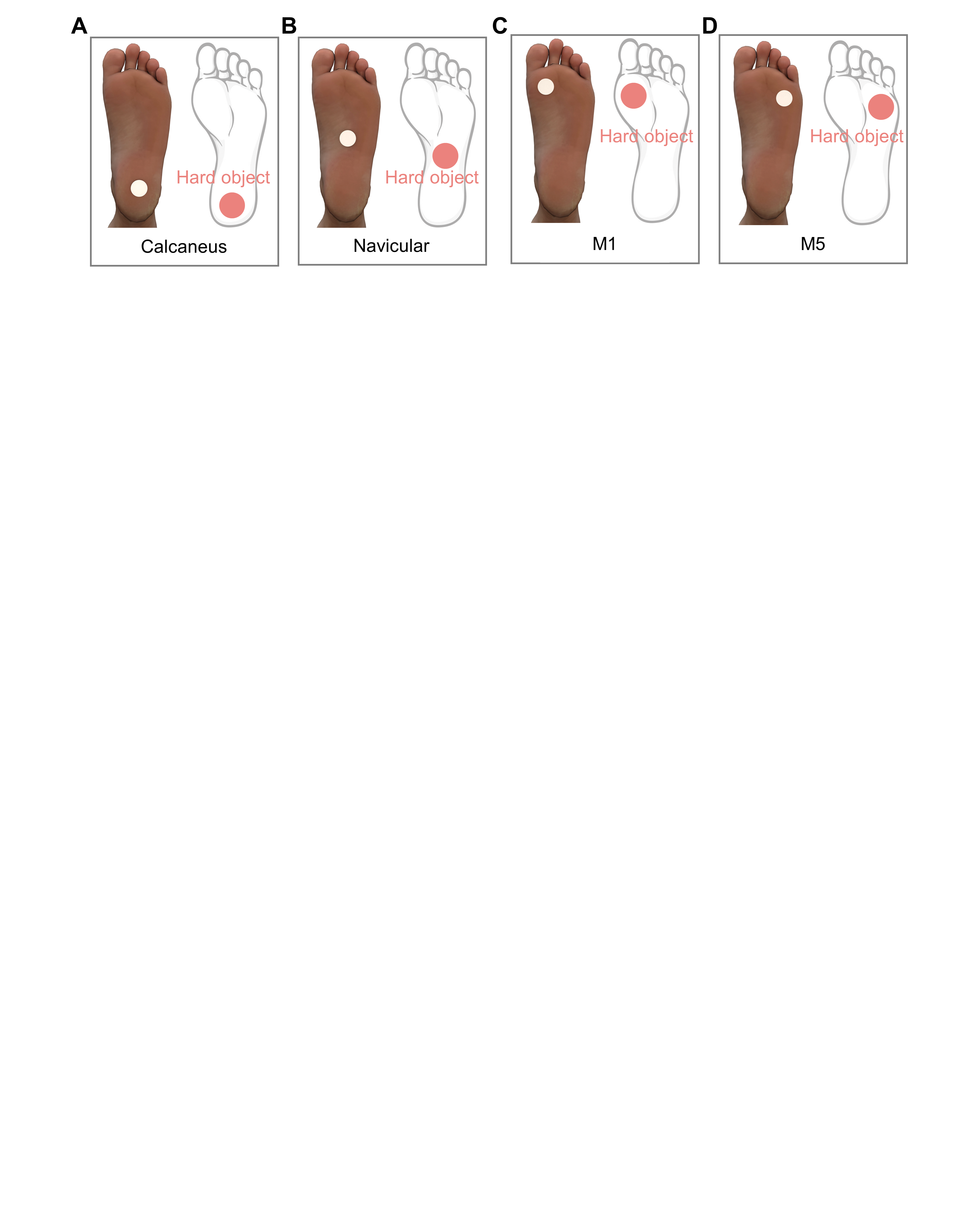


**Fig. S31. Different types of plantar pressure distributions for classification.** The round areas are hard blocks used to create abnormal stress points.


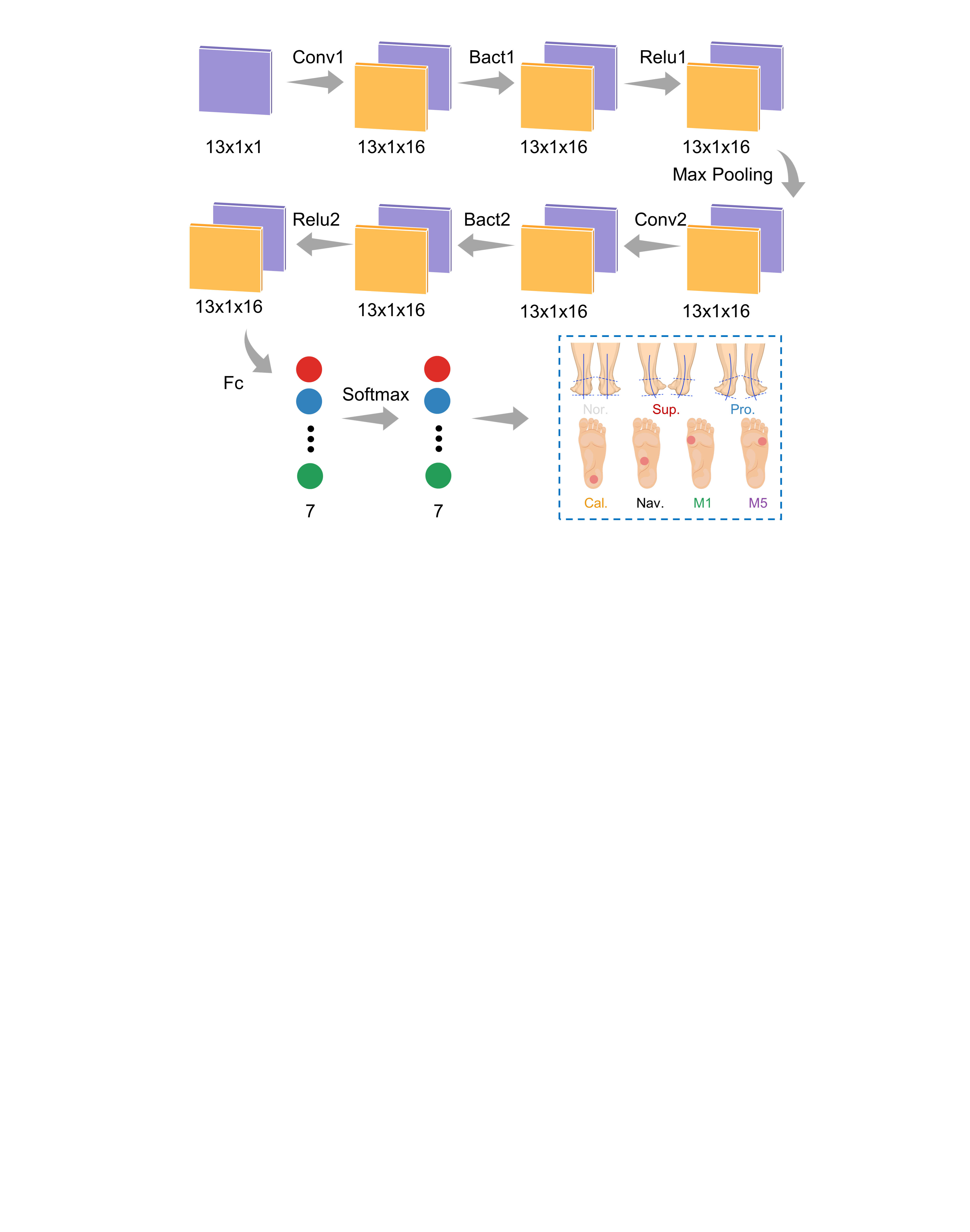


**Fig. S32. Schematic diagram of CNN network architecture to detect abnormalities.** The architecture illustrates the layer-wise flow from the input pressure signals (13×1) to the final classification output.


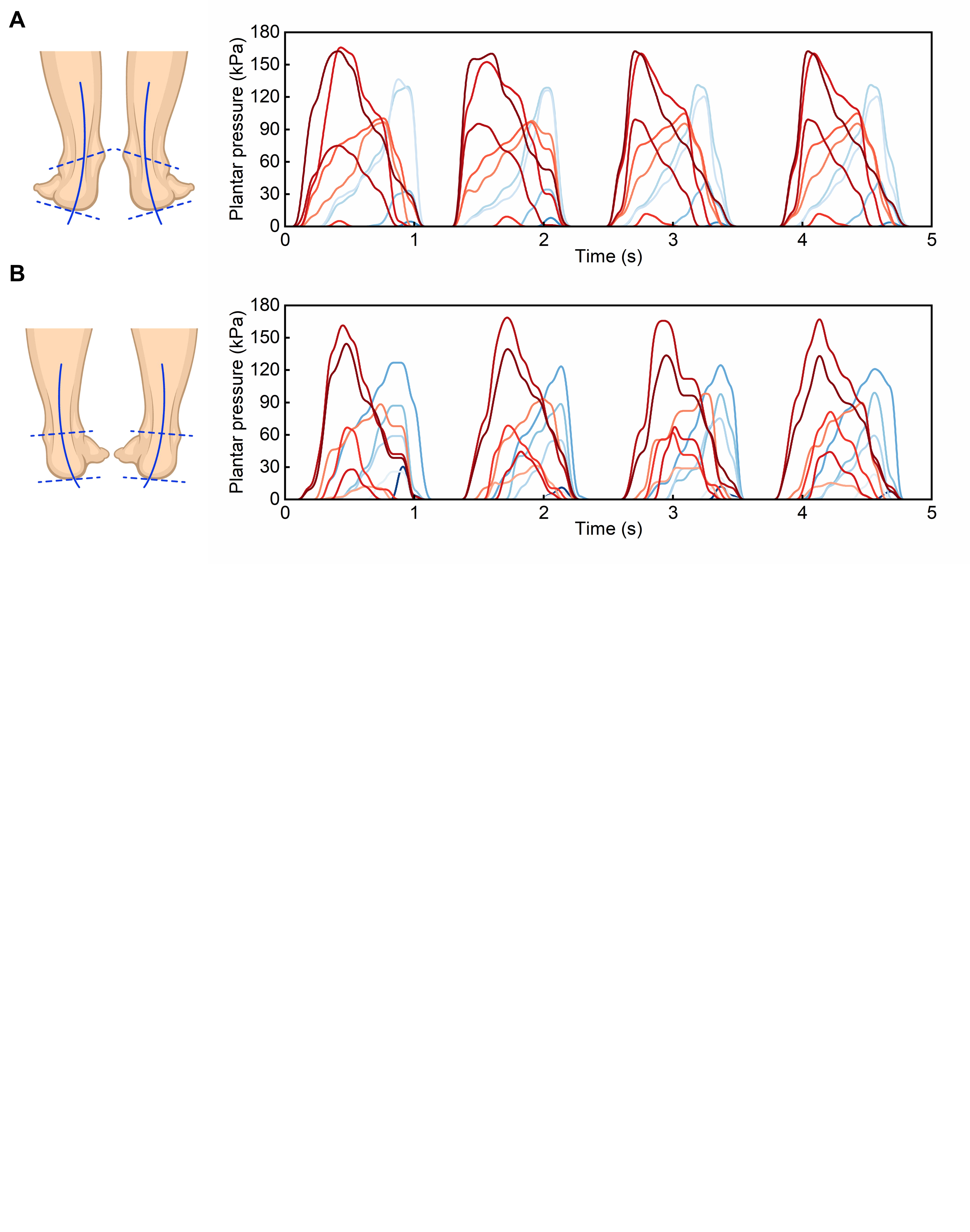


**Fig. S33. Abnormal gait data used to train the CNN model.** (**A**) Pressure data of supinator. (**B**) Pressure data of pronator.


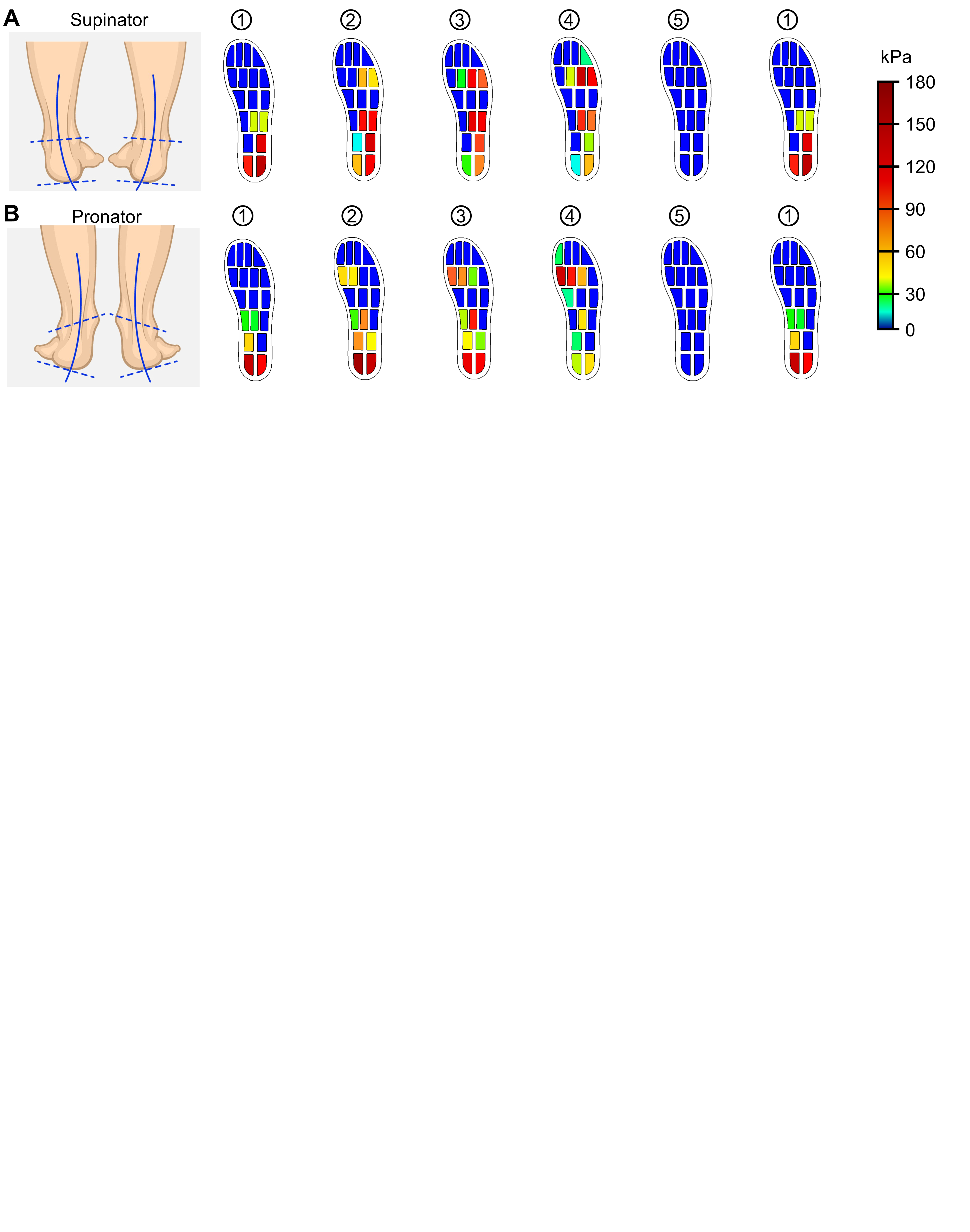


**Fig. S34.** **Thermographic maps of abnormal gaits.** (**A**) Pressure maps of supinator. (**B**) Pressure maps of pronator.


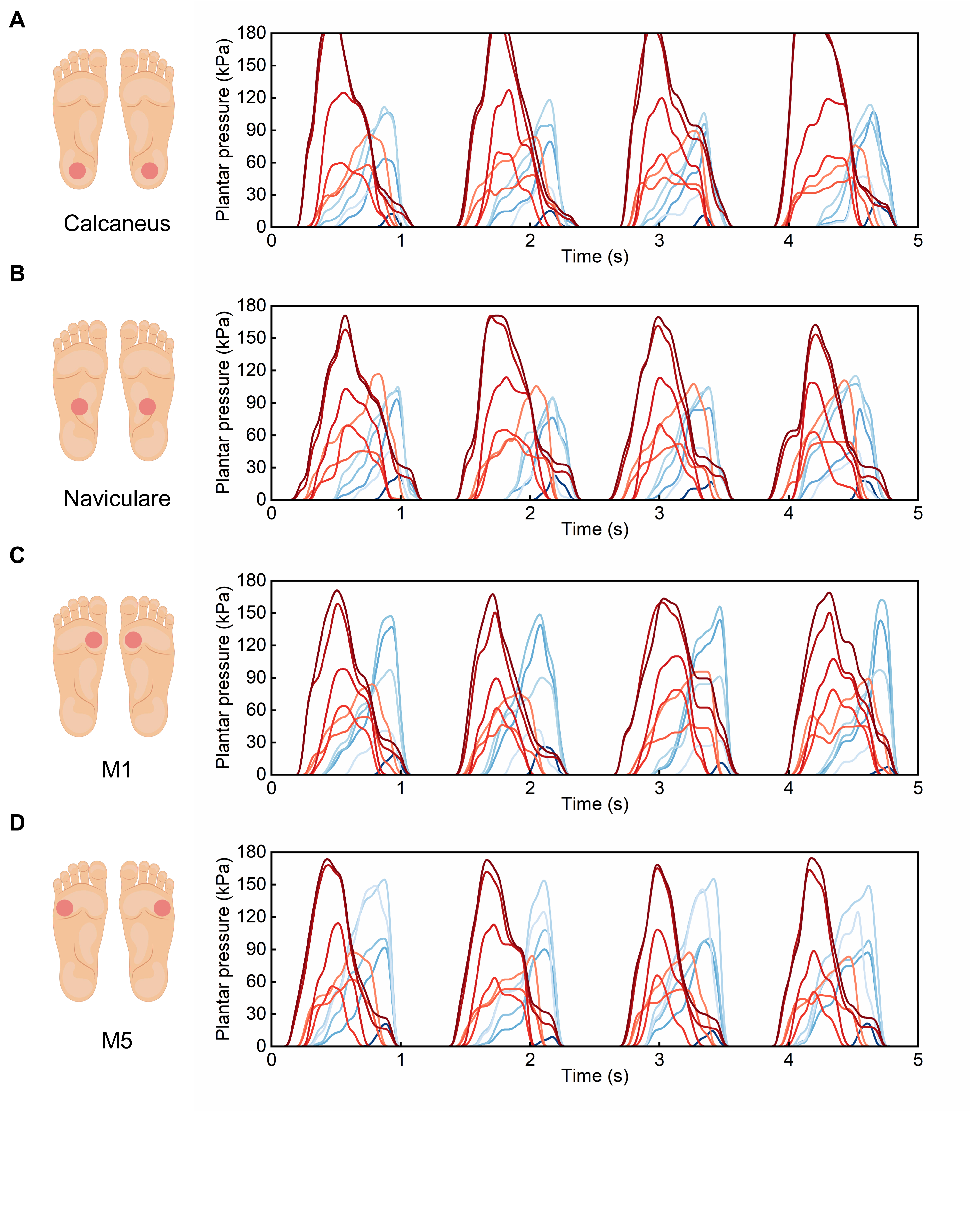


**Fig. S35. Abnormal pressure point data used to train the CNN model.** (**A**) Data of abnormal calcaneus pressure. (**B**) Data of abnormal naviculare pressure. (**C**) Data of abnormal first metatarsal pressure. (**D**) Data of abnormal fifth metatarsal pressure.


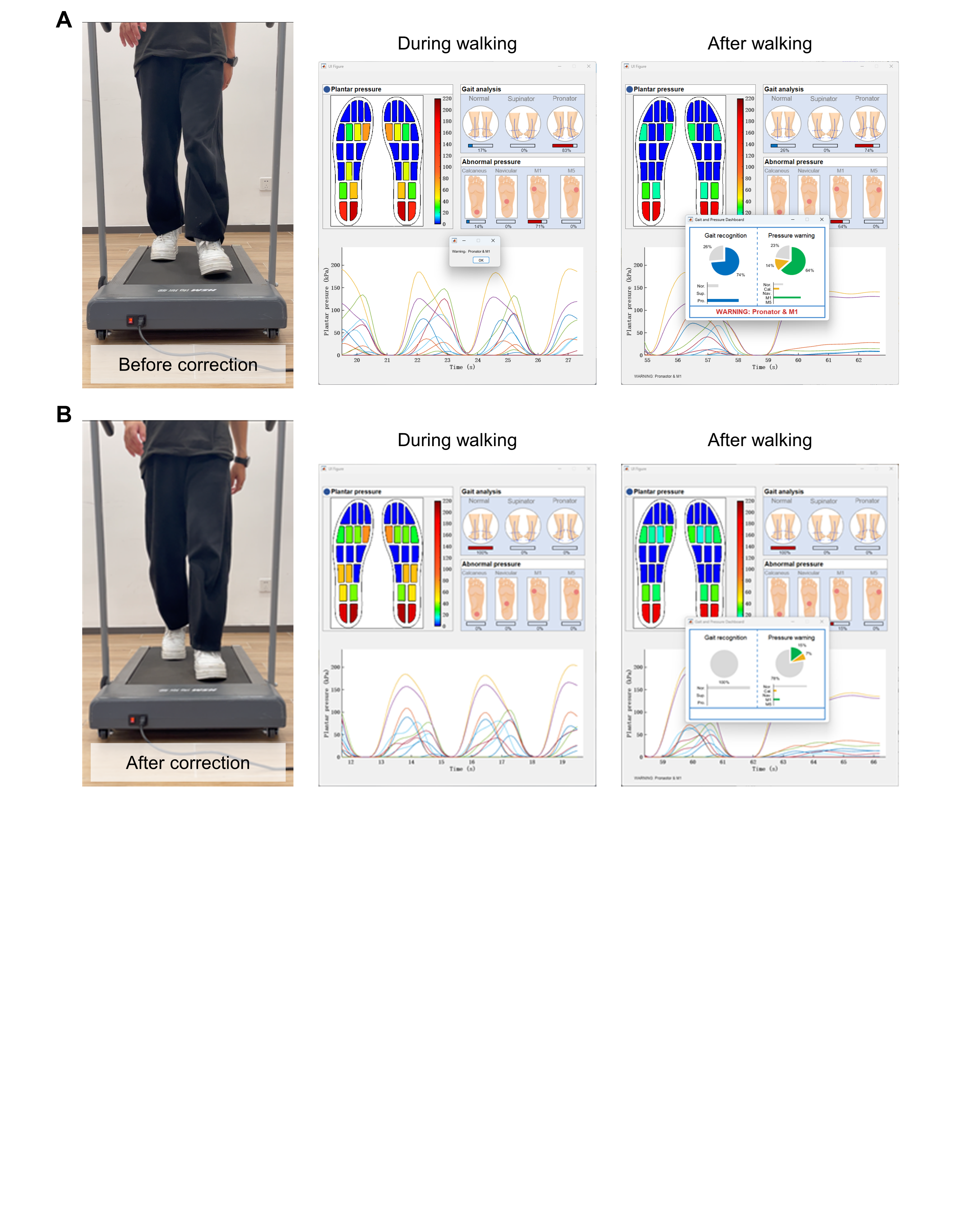


**Fig. S36. Real-time monitoring and feedback for active gait correction.** (**A**) Before correction. (**B**) After correction. When the user exhibits abnormal gait during walking, the VFS system will promptly issue an alert warning and, after the gait cycle is completed, provide a gait report for self-inspection. After the user's gait has been corrected, by referring to the gait report provided by the VFS system, it can be observed that the abnormal gait has significantly decreased. Therefore, the experiment has proved that VFS can help users effectively correct their gait.

**Supplementary Tables**

**Table S1. Specification of system components and source breakdown.**

| **Module** | **Component** | **Description** | **Type** |
| --- | --- | --- | --- |
| ASA | Transmission mechanism | Ball screw & gear system | Self-developed & machined |
|  | Generator | Brushless motor | Commercial |
|  | Structural shell | Frame | Self-developed & machined |
|  | Compression spring | Stiffness: 10 N/mm | Customized selection |
| WSS | Pressure sensors | Flexible resistive sensor array | Commercial |
|  | MCU & Bluetooth | Data processing & transmission | Customized selection |
| VFS | Gait recognition | CNN-based classifier | Self-developed algorithm |
|  | User interface | Program | Self-developed software |

**Table S2. Summary of material parameters of the model.**

| **Description** | **Density (kg/m^3^)** | **Young’s modulus (kPa)** | **Poisson’s ratio** |
| --- | --- | --- | --- |
| Bone | 1500 | 7300 | 0. 3 |
| Soft tissue | 937 | 0. 45 | 0. 49 |
| Insole | 500 | 2 | 0. 35 |
| Shoe sole | 5000 | 17000 | 0. 1 |
| ASA’ shell | 5000 | 17000 | 0. 1 |
| ASA’ spring | 1000 | 1. 5 | 0. 35 |

**Table S3. Geometric parameters of components of the ASA prototype.**

| **Description** | **Symbol** | **Value** | **Supplier** |
| --- | --- | --- | --- |
| Length of lead screw | *L*_1_ | 38.5 mm | Billionyi bearing Co. , Ltd |
| Pitch of lead screw | *p* | 10 mm | Billionyi bearing Co. , Ltd |
| Diameter of lead screw | *D*_1_ | 8 mm | Billionyi bearing Co. , Ltd |
| Diameter of the ball | *D*_2_ | 1.2 mm | Billionyi bearing Co. , Ltd |
| Outside diameter of the nut | *D*_3_ | 18 mm | Billionyi bearing Co. , Ltd |
| Thickness of sleeve | *σ* | 5 mm | Machining |
| Height of the sleeve | *H* | 30 mm | Machining |
| Stiffness of spring | *K* | 10 N/mm | Chuang exhibition trade Co. , LTD |
| Length of spring | *L*_2_ | 25, 35, 45 mm | Chuang exhibition trade Co. , LTD |
| Radius of motor | *R* | 28.5 mm | Youweike Technology Co. , Ltd |
| Thick of the baseboard | *L*_3_ | 3.5 mm | Machining |
| Length of the gearbox | *L*_4_ | 65 mm | Assembled |
| Width of the gearbox | *L*_5_ | 35 mm | Assembled |

**Table S4. Overall geometric dimensions of the ASA prototype.**

| **Description** | **Symbol** | **Value** | **Note** |
| --- | --- | --- | --- |
| Length of the device | *l* | 100 mm | Assembled |
| Height of the device’s outer frame | *h*_1_ | 48 mm | Assembled |
| Maximum height of the device | *h*_2_ | 60 mm | Assembled |
| Width of the device | *w* | 40 mm | Assembled |
| Travel distance of the sleeve in a gait | *h*_3_ | 10 mm | At the stance phase |
| Total mass of the device | *m* | 248±1 gram | Fluctuation with spring stiffness |
| Total volume of the device | *V* | 125 cm^3^ | Assembled |

**Table S5. Materials of components of the ASA prototype.**

| **Component** | **Material** |
| --- | --- |
| Lead screw | SF53 stainless steel |
| Nut | 20CrMo alloy structural steel |
| Spring | Stainless steel |
| Gear | Polyoxymethylene |
| Electrode | Copper |
| Sleeve | Acrylic |
| Baseboard | 6061 Aluminum alloy |
| Shell | Acrylic |

**Table S6. Comparison of the proposed ASA device with the reported foot energy harvesters.**

| **Ref.** | **Source** | **Mode** | **Mass (g)** | **Volume (cm^3^)** | **Power (mW)** | **Den. 1**  **(mW/g)** | **Den. 2**  **(mW/cm^3^)** |
| --- | --- | --- | --- | --- | --- | --- | --- |
| [1] | Shoe | Ele. | 120 | 80* | 11 | 0.09 | 0.13 |
| [2] | Sole | Pie. | 12.5 | 165 | 34 | 2.72 | 0.20 |
| [3] | Shoe | Ele. | 22 | 48 | 4.1 | 0.18 | 0.08 |
| [4] | Sole | Ele. | 200* | 103 | 1000 | 5.00 | 9.70 |
| [5] | Sole | Mag. | 340 | 40* | 80 | 0.13 | 2.00 |
| [6] | Sole | Ele. | 18.6 | 6.47 | 2.15 | 0.11 | 0.33 |
| [7] | Sole | Pie. | 226 | 153 | 8.5 | 0.03 | 0.05 |
| [8] | Sole | Pie. | 105 | 46 | 0.38 | 3.61*10^-3^ | 8.26*10^-3^ |
| **This work** | **Sole** | **Ele.** | **248** | **125** | **5200** | **20.00** | **41.60** |

*** Estimated based on device.**

**Electromagnetic (Ele.), Piezoelectric (Pie.), Magnetofluid (Mag.).**

**Note: The proposed ASA device exhibits the highest power density (41.60 mW/cm³) among the listed electromagnetic, piezoelectric, and magnetofluid harvesters, validating its efficiency for self-powered applications.**

**Table S7. Multiple gait plantar pressure.**

| **Gait**  **Region** | | **Standing** | **Jogging** | **Upstairs** | **Downstairs** | **Upslope** | **Downslope** |
| --- | --- | --- | --- | --- | --- | --- | --- |
| **Calcaneus**  **(kPa)** | Without device | 102.39 | 311.47 | 152.84 | 166.23 | 208.55 | 174.79 |
|  | With device | 76.67 | 210.62 | 115.18 | 83.8 | 125.32 | 147.78 |
| **M1**  **(kPa)** | Without device | 30.75 | 93.94 | 59.42 | 142.55 | 68.65 | 78.88 |
|  | With device | 29.6 | 87.68 | 54.3 | 129.53 | 66.95 | 55.37 |
| **M5**  **(kPa)** | Without device | 20.29 | 87.11 | 51.23 | 90.46 | 71.93 | 92.73 |
|  | With device | 19.94 | 82.48 | 51.49 | 100.39 | 65.73 | 72.12 |
| **Phalanx**  **(kPa)** | Without device | 2.13 | 60.74 | 13.13 | 23.92 | 14.72 | 20.05 |
|  | With device | 2.16 | 57.31 | 12.16 | 33.39 | 12.12 | 29.94 |
| **Navicular**  **(kPa)** | Without device | 0 | 13 | 0 | 0 | 7.14 | 0 |
|  | With device | 24.51 | 105.59 | 60.34 | 69.35 | 76.23 | 56.07 |

**Table S8. Detailed parameters of the CNN algorithm model for gait recognition.**

| **No.** | **Layer type** | **No. of filters** | **Pool size** | **Stride** | **Input size** | **Output size** |
| --- | --- | --- | --- | --- | --- | --- |
| 1 | Input 1 |  |  |  | 13×1×1 | 13×1×1 |
| 2 | Convolution 1 | 16 | 2×1×16 | (1, 1) | 13×1×1 | 13×1×16 |
| 3 | Batch Normalization 1 | 16 |  |  | 13×1×16 | 13×1×16 |
| 4 | ReLU 1 |  |  |  | 13×1×16 | 13×1×16 |
| 5 | Max Pooling |  | 2×1 | (2, 1) | 13×1×16 | 6×1×16 |
| 6 | Convolution 2 | 32 | 2×1×32 | (2, 1) | 6×1×16 | 6×1×32 |
| 7 | Batch Normalization 2 | 32 |  |  | 6×1×32 | 6×1×32 |
| 8 | ReLU 2 |  |  |  | 6×1×32 | 6×1×32 |
| 9 | Fully Connected |  |  |  | 192 | 7 |
| 10 | Softmax |  |  |  | 7 | 7 |
| 11 | Classification |  |  |  | 7 | 7 |

**Table S9. Participant characteristics.**

| **Participant** | **Height (m)** | **Body mass (kg)** | **Age (years)** | **Experiment order*** |
| --- | --- | --- | --- | --- |
| 1 | 1.79 | 62 | 24 | Yes → No |
| 2 | 1.65 | 59 | 24 | Yes → No |
| 3 | 1.73 | 74 | 29 | No → Yes |
| 4 | 1.75 | 68 | 26 | No → Yes |
| 5 | 1.83 | 78 | 23 | No → Yes |
| 6 | 1.69 | 54 | 25 | Yes → No |
| 7 | 1.72 | 72 | 28 | Yes → No |
| Mean | 1.74±0.05 | 67±2 | 25.5±0.5 |  |

*** Yes=with device; No=without device**

**Supplementary Texts**

**Text S1. Inverted pendulum model**

The impact force on the foot is determined by several factors, such as body weight, ground contact mechanics, and footwear conditions. The time integral of the impact force equals the change in momentum of the body as the foot suddenly decelerates while the rest of the body gradually slows down. This process is equivalent to the sudden stoppage of the effective body mass of the center of mass (*m_eff_*) during a foot-ground collision. The relationship between the collision and momentum change can be expressed as:

where *T* is the duration of the touchdown moment; *F_z_*(*t*) is the resisting force of the plantar deformation; *z* is the vertical displacement of the foot’s deformation; *g* is the vertical displacement of the foot’s deformation. The superscript symbolizes the first derivation with respect to time *t*.

Using the integral median theorem, **Eq. S1** can be written as:

For the stiffness *k* of the ASA prototype, it can be expressed as:

where $\Delta x$ is the compression of the spring at touchdown. The stiffness of the ASA device is not only related to the effective body mass but is also influenced by the transient impact forces associated with the ground contact torque. To determine the appropriate spring stiffness, biomechanical simulations are conducted for the initial ground contact phase (0–21% of the gait cycle).

The purpose of the simulation is to predict the required support force for the arch and to determine the optimal spring stiffness for the ASA, as well as to study the interaction between biomechanics and the ASA under dynamic loading conditions. To simplify the analysis of the ASA’s influence during human walking, the ASA is divided into two components: the ASA shell and the ASA spring. The ASA shell is modeled as a rigid material, while the ASA spring is modeled as a viscoelastic material. A finite element model of the foot is established based on participants aged 20–30 years with a body weight of 65 kg wearing the ASA. Additionally, asymmetrical ground contact conditions are considered, while air resistance during slow walking is neglected. The material parameters and structural details used in this model are presented in **tables S2-S5** and **fig. S9**.

In this model, lumped parameters are used to define the inertial and motion characteristics of body mass. Considering the small displacement between the foot, the insole, the ASA, and the shoe sole are bound together to form the shoe, while the soft tissue and bones are bound together to form the foot. The foot is rotated 5° to make contact with the sole. For a body weight of 65 kg (under slow walking conditions), the boundary load is applied to the tibia, ranging from 0 to 300 N over 0.21 seconds (with the entire stride lasting one second). Based on the reduction in plantar stress during walking, the required support pressure for the arch is estimated to be approximately 80–100 kPa (**fig. S10)**. As a result, a series of springs is tested experimentally to identify the optimal configuration for the ASA design. The selection of spring lengths (25 mm, 35 mm, and 45 mm) is determined based on the geometric constraints of the shoe heel and the biomechanical requirement for adjustable support intensity. Given the minimum thrust required for device reset, with the spring installation space fixed at approximately 10 mm, the 25 mm spring represents the baseline condition with initial preload (soft support), and the 35 mm and 45 mm springs introduce 25 and 35 mm of pre-compression, respectively. This range effectively covers the transition from compliant cushioning to rigid support, allowing for the empirical validation of the optimal physiological pressure range (80–100 kPa) predicted by the simulation.

**Text S2. The voltage output of the energy harvester**

To characterize the dynamic response of the energy harvester, the prototype is simplified into a unidirectional coupled model, considering only the effect of mechanical responses on electrical responses. Under external excitation, the sleeve and nut move downward simultaneously, and the screw rotates under the constraint of the threaded guide rail. Based on a single-degree-of-freedom spring-mass-damping system, the motion equations for the sleeve and nut under external excitation are as follows:

where *F* represents the external excitation, *m* is the mass of the sleeve and nut, *k* is the stiffness of the compression spring, *c* is the equivalent damping constant, *z* is the number of threads, *f_N_* is the normal force acting on the single-threaded screw, *f_r_* is the frictional force acting on the single-threaded screw, *φ* is the helix angle, *x*(*t*) is the vibration amplitude of the nut and sleeve (**fig. S4**).

The translational vibration of the sleeve and the rotational motion of the lead screw are related by:

$$x(t)=\frac{\theta}{2\pi}p$$

where *θ* denotes the rotation angle of the screw, and *p* is the pitch of the sleeve.

Next, based on Faraday’s law of electromagnetic induction, the electrical response of the energy harvesting device can be derived,

$$V(t)=-N\frac{d}{dt}[BS\cos(\omega t)]=NBS\omega\sin(\omega t)$$

where *N* is the number of turns in the coil wrapping around the rotor, *B* is the density of the magnetic field, *S* is the area of the coils, and *ω* is the angular velocity of the electromagnetic rotor.

For the four gear pairs (shown in **fig. S2**), the number of teeth on the gears can be represented as *Z_i_*_1_, *Z_i_*_2_, respectively, and *i* = 1, 2, 3, 4, then the total transmission ratio *K* of the gearbox can be expressed as:

$$K=\prod_{i=1}^{4} \frac{Z_{i1}}{Z_{i2}}$$

Thus, the angular velocity of the rotor is

$$\omega=\frac{2\pi\times x(t)}{Pt}\prod_{i=1}^{4} \frac{Z_{i1}}{Z_{i2}}$$

Considering the relation , we obtain,

$$V(t)=\frac{2\pi NBSKx(t)}{Pt}\sin\left(\frac{2K\pi x(t)}{P}\right)$$

The current in the coil can be expressed as:

$$I(t)=\frac{V(t)}{R_{C}+R_{L}}$$

where *R_c_* represents the total resistance of the coil, and *R_L_* denotes the external load resistance. Thus, the power output *P*(*t*) of the energy harvesting device is given by:

$$P(t)=\frac{V(t)^{2}}{R_{L}}$$

The above indicates that the performance of the energy harvesting device is determined by the relative displacement between the nut and the heel base, the pitch of the nut, and the intrinsic characteristics of the electromagnetic generator. Once the size and physical properties of the energy harvesting device are fixed, the damping ratio becomes the key factor influencing the system’s output. To obtain the open-circuit voltage under different gait patterns and validate the frequency conversion mechanism, tests are conducted on a 24-year-old male participant (weighing 65 kg).

**Text S3. Circuit power consumption calculation**

To quantify the energy demand, we evaluate the WSS consumption (including sensing, MCU, and Bluetooth) via the capacitor discharge method. The average power (*P*_avg_) is derived from the energy change (Δ*E*) over duration (Δ*t*):

$$P_{avg}=\frac{C(V_{start}^{2}-V_{end}^{2})}{2\Delta t}$$

Based on empirical data from Movie S3, with *V_start_* = 42 V, *V_end_* = 22 V, Δ*t* = 27 s, and *C* = 6800 μF, the system consumed approximately 161 mW. This energy budget is well-covered by the ASA’s output (>200 mW), ensuring a reliable safety margin for continuous operation.

**Other Supplementary Materials for this manuscript include the following:**

Movie S1 (.mp4 format). Demonstration of lighting up two 3 W LED bulbs.

Movie S2 (.mp4 format). Powering a commercial watch.

Movie S3 (.mp4 format). Powering a wearable sensing system.

Movie S4(.mp4 format). Fully self-powered wearable system for real-time and visualized plantar pressure monitoring.

Movie S5 (.mp4 format). Closed-loop real-time monitoring and feedback for active gait correction through the VFS system.

**References**

[1] W. Wang, J. Cao, N. Zhang, J. Lin, W.-H. Liao, Magnetic-spring based energy harvesting from human motions: Design, modeling and experiments, *Energy Conversion and Management* **2017**, *132*, 189.

[2] S. Wen, Z. Wu, Q. Xu, Design of a Novel Two-Directional Piezoelectric Energy Harvester With Permanent Magnets and Multistage Force Amplifier, *IEEE Transactions On Ultrasonics, Ferroelectrics, and Frequency Control* **2020**, *67*, 840.

[3] K. Ylli, D. Hoffmann, A. Willmann, P. Becker, B. Folkmer, Y. Manoli, Energy harvesting from human motion: exploiting swing and shock excitations, *Smart Mater. Struct.* **2015**, *24*, 25029.

[4] L. Xie, M. Cai, An In-Shoe Harvester With Motion Magnification for Scavenging Energy From Human Foot Strike, *Ieee/asme Trans. Mechatron.* **2015**, *20*, 3264.

[5] D. Dai, J. Liu, Y. Zhou, Harvesting biomechanical energy in the walking by shoe based on liquid metal magnetohydrodynamics, *Front. Energy* **2012**, *6*, 112.

[6] M. A. Halim, H. Cho, J. Y. Park, Design and experiment of a human-limb driven, frequency up-converted electromagnetic energy harvester, *Energy Conversion and Management* **2015**, *106*, 393.

[7] F. Qian, T.-B. Xu, L. Zuo, Material equivalence, modeling and experimental validation of a piezoelectric boot energy harvester, *Smart Mater. Struct.* **2019**, *28*, 75018.

[8] L. Xie, M. Cai, Increased piezoelectric energy harvesting from human footstep motion by using an amplification mechanism, *Applied Physics Letters* **2014**, *105*.
